# The Application of 2D Graphitic Carbon Nitride (g-C_3_N_4_) and Hexagonal Boron Nitride (h-BN) in Low-Temperature Fuel Cells: Catalyst Supports, ORR Catalysts, and Membrane Fillers

**DOI:** 10.3390/molecules30081852

**Published:** 2025-04-20

**Authors:** Ermete Antolini

**Affiliations:** Scuola di Scienza dei Materiali, Via 25 Aprile 22, Cogoleto, 16016 Genova, Italy; ermantol@libero.it

**Keywords:** g-C_3_N_4_, h-BN, fuel cells, catalysts, polymer membranes

## Abstract

In recent years, two-dimensional (2D) graphitic carbon nitride (g-C_3_N_4_) and hexagonal boron nitride (h-BN) have gained remarkable attention due to their resemblance to graphene. These materials have a wide range of applications in energy and other sustainable fields, including heterogeneous catalysis and photocatalysis. g-C_3_N_4_ and h-BN can play different roles in low-temperature fuel cells. They can be used as catalyst supports, catalysts for oxygen reduction, and membrane fillers. In this work, the application of pure and doped g-C_3_N_4_ and h-BN, alone or as composite materials, in low-temperature fuel cells is overviewed.

## 1. Introduction

**Scheme 1:**
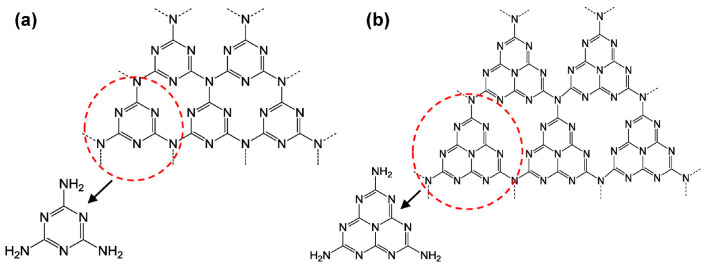
(**a**) Triazine and (**b**) tri-s-triazine (heptazine) structures of g-C_3_N_4_. In these g-C_3_N_4_ structures, different nitrogen species (pyridine, amine, imine, and quaternary nitrogen) are present. (**a**) red dotted circle: triazine unit; (**b**) red dotted circle: heptazine unit. Reproduced from Ref. [1], copyright 2024, with permission from Elsevier.

Two-dimensional carbon nitride and boron nitride are promising and widely used materials. Recently, graphitic carbon nitride (g-C_3_N_4_) and hexagonal boron nitride (h-BN) compounds attracted remarkable attention related to their resemblance to graphene [1,2]. In g-C_3_N_4_, C and N atoms adopt sp2 hybridization and are interconnected through σ bonds. The hexagonal structure comprises triazine rings, or hexatomic rings, which are attached to a smaller unit σ through C-N bonds. There exist two chemical structures for g-C_3_N_4_, featuring triazine rings (C_3_N_3_) and tri-s-triazine rings (C_6_N_7_). Scheme 1 shows the structures of triazine and tri-s-triazine.

Due to the high nitrogen-to-carbon ratio, g-C_3_N_4_ is appropriate for applications where nitrogen-rich compounds are critical. Its unique structure gives outstanding stability to the material versus thermal and chemical attacks. By doping or incorporating it with other compounds, the characteristics of g-C_3_N_4_ can be easily tailored. Thus, it is possible to obtain g-C_3_N_4_ compounds with various morphologies, improving their performance and extending the range of applications in energy and other sustainable fields, including heterogeneous catalysis and photocatalysis [1].

**Scheme 2:**
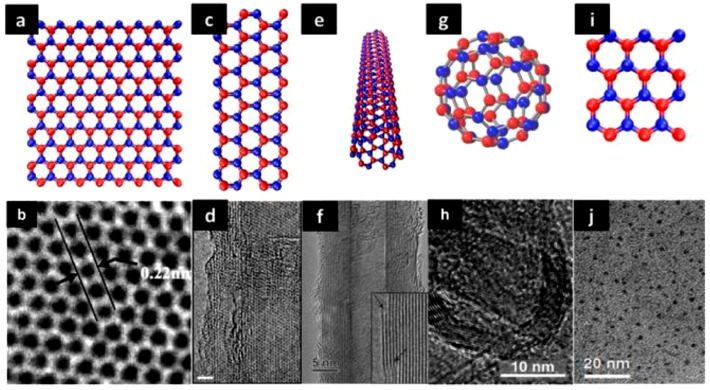
Schematic representation of h-BN nanomaterials: (**a**,**b**) h-BN nanosheets (h-BNNSs); (**c**,**d**) h-BN nanoribbons (h-BNNRs); (**e**,**f**) h-BN nanotubes (h-BNNTs); (**g**,**h**) h-BN fullerenes (h-BNFLs); (**i**) h-BN quantum dots (h-BNQDs). Reproduced from Ref. [2], copyright 2022, with permission from Elsevier.

Boron nitride materials have attracted increasing interest in relation to their high electrical resistance and thermal conductivity, good chemical inertness and thermal shock resistance, and low thermal expansion [2]. Among the different crystalline forms of BN, h-BN is especially intriguing as an emerging 2D material because of its exceptional properties. h-BN is a layered compound similar to graphite, composed of alternating sp^2^-bonded B and N atoms, held together by covalent bonds, and the layers are held together by van der Waals forces [2]. The outstanding properties of BN-based materials make them attractive for applications in the fields of energy storage and health care.

h-BN nanomaterials can be found in all morphological shapes, such as nanosheets, nanoribbons, nanotubes, fullerenes, and quantum dots (Scheme 2). Hexagonal boron nitride nanosheets (h-BNNSs) are one of the more interesting nanoforms of BN, which resemble graphene nanosheets. BNNS has a 2D morphology like graphene and may have a thickness of 0.35–100 nm. BNNSs have excellent mechanical properties and thermal conductivity, high thermal stability, and oxidation resistance. Owing to their properties, the exfoliation of 2D h-BNNSs from bulk h-BN materials has received growing interest. Numerous exfoliation techniques offer scalable approaches for harvesting single-layer or few-layer h-BNNSs. Exfoliation from bulk-stacked h-BN is the most cost-effective way to obtain large quantities of few-layered h-BN.

**Figure 1:**
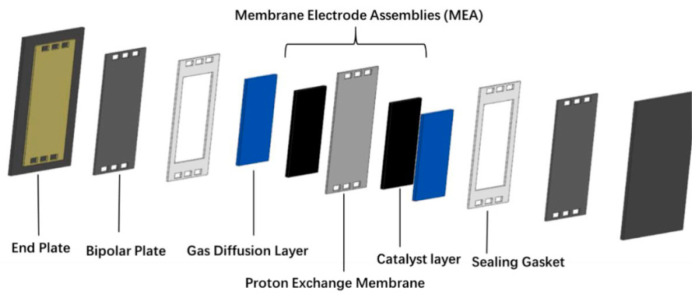
Layout of a PEMFC. Reproduced from Ref. [4], copyright 2023, with permission from Elsevier.

**Scheme 3:**
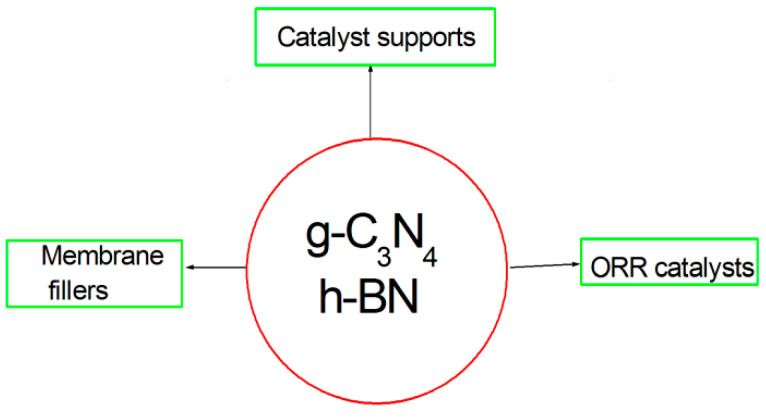
The use of g-C_3_N_4_ and h-BN in low-temperature fuel cells.

A polymer electrolyte membrane fuel cell is essentially composed of the membrane electrode assembly (MEA) and the bipolar plates (BPs) [3,4]. The MEA includes electrodes formed by a gas diffusion layer (GDL) and a catalyst layer (CL)—the main component of which is a supported catalyst—on the anode and cathode side, respectively, and a proton exchange membrane (PEM) or an anion exchange membrane (AEM) in between [3]. A schematic view of the proton exchange membrane fuel cell (PEMFC) structure is shown in Figure 1. Two-dimensional nitrides are promising materials that can be used either as catalyst supports—having higher stability in PEMFC environments than the commonly used carbon supports and improving the catalytic activity of the supported catalysts by catalyst-support interactions—or as catalysts for the oxygen reduction reaction (ORR), replacing noble metal catalysts and showing good catalytic activity [5]. More recently, due to the layered structure with regular triangular nanopores acting as ion transport channels for H_2_O molecules and protons, g-C_3_N_4_ and h-BN have been used in polymer electrolyte membranes. The use of g-C_3_N_4_ and h-BN in low-temperature fuel cells is shown in Scheme 3. In this work, the feasible use of 2D nitrides as catalyst supports, catalysts for the ORR, and membrane fillers in low-temperature fuel cells is overviewed.

## 2. Catalyst Supports

Carbon is typically used as a catalyst support in polymer electrolyte membrane fuel cells, related to its large surface area, high electrical conductivity, and low price [6]. However, carbon particles are electrochemically oxidized under PEMFC operating conditions. The low stability of carbon support leads to a decrease in Pt surface area due to both Pt particle sintering and Pt loss [6]. Corrosion mainly affects the cathode catalyst support, but the anode catalyst support could also be prone to oxidative conditions [7]. Moreover, Pt catalysts accelerate the rate of carbon corrosion [8]. Degradation of the support also leads to a lowering of the rate of mass transport. For these reasons, finding new types of catalyst supports with high corrosion resistance in a fuel cell environment is mandatory. Various advanced carbon materials, such as ordered mesoporous carbons, nanostructured 1D carbons, and graphene, have been proposed [6,9], but these materials only decrease the carbon oxidation rate. Therefore, non-carbon materials, such as transition metal oxides, carbides, and nitrides [5,10,11], were evaluated as catalyst supports. Moreover, some of them can act as co-catalysts [10]. Its lower price, excellent mechanical resistance, and high chemical stability at high temperatures and in acid and basic environments make g-C_3_N_4_ suitable for use as a fuel cell catalyst support [12,13]. A problem regarding the utilization of g-C_3_N_4_ as a fuel cell catalyst support is its poor conductivity. Moreover, its low surface area still hinders its application as a fuel cell catalyst support. A way to increase conductivity is the addition of the g-C_3_N_4_ of a conductive compound, forming a composite material [14]. In this regard, g-C_3_N_4_ can be divided into two classes: bare g-C_3_N_4_ supports and composite g-C_3_N_4_/carbon supports. Regarding the latter, generally, g-C_3_N_4_/carbon composites act as true hybrid supports with improved properties, such as higher electron conductivity (by the presence of carbon) or higher corrosion resistance (by the presence of the nitride) than the single materials. In some cases, the carbon-based material only acts as a support for g-C_3_N_4_ to increase its surface area. In recent works, 2D h-BN has also been investigated as a fuel cell catalyst support, but only for out-of-cell tests.

### 2.1. Carbon Nitrides

It is hopeful that nitrogen-doped carbons can act as catalyst supports because the coupling of the carbon π-system and the nitrogen electron lone pair has a considerable effect on carbon electronic properties [15]. N doping creates active sites on the carbon surface, acting as an anchoring site for the catalyst nanoparticles, resulting in an improved dispersity and stability of the catalyst [16]. Cai et al. [17] observed that Pt dispersion increases with increasing N doping of the carbon support. N-doped carbon can be obtained either by the calcination of metal-organic frameworks (MOFs) or by carbon treatment with N precursors at elevated temperatures. These approaches, however, provide little or no control over porous structures, particle morphologies, sizes, and nitrogen states; this can be a drawback, as certain functionalities, such as pyridinic N, contribute to the enhancement of catalytic activity. Conflicting results on electrochemical corrosion were reported. Brandiele et al. [18] found that N doping improves carbon corrosion resistance. Conversely, in a recent work, Homberger et al. [19] observed that N doping and, thus, the incorporation of defects into the carbon backbone do not reduce carbon corrosion during high-potential cycling (1–1.5 V). The atomic structure of graphene is disrupted by the presence of free nitrogen atoms [20]. The excess of N doping reduces carbon corrosion resistance due to the existence of free nitrogen atoms. Thus, it is mandatory to carefully control the amount of N doping on carbon materials to avoid a decrease in corrosion resistance. To overcome the drawbacks of poor structural control during synthesis and the corrosion of nitrogen-doped carbons, carbon nitrides are considered hopeful candidates as fuel cell catalyst support. Indeed, carbon nitrides supply a high amount of N-specific functionalities at definite positions. Moreover, the high corrosion resistance and chemical stability make them promising catalyst supports. Among carbon nitride materials, 2D g-C_3_N_4_ is the most stable, with graphite-like planes [12,13].

#### 2.1.1. Pristine g-C_3_N_4_

Due to the presence of N atoms, carbon nitrides supply a suitable bonding environment to anchor catalyst particles. The triazine/metal coordination complex enhances catalyst durability [13]. Due to its 2D layered structure, g-C_3_N_4_ can also provide a higher surface area than the bulk material and, therefore, can support a higher amount of catalyst. Mansor et al. [12] explained the enhanced chemical resistance to corrosion of g-C_3_N_4_ compared to carbon by using simple band structure considerations. First, Kim et al. [21] used g-C_3_N_4_ with highly ordered pore arrays as a support for PtRu. A direct methanol fuel cell (DMFC) with PtRu/g-C_3_N_4_ as the anode catalyst delivered a maximum power density (MPD) of ca. 83% higher than that with PtRu/C. Moreover, the MPD of the DMFC with a PtRu/C_3_N_4_ anode was ca. 25% higher than that with an ordered mesoporous carbon (OMC)-supported PtRu catalyst. Yue et al. [22], Ma et al. [23], and Sadhukhan et al. [24] deposited Pt nanoparticles on 1D g-C_3_N_4_ nanotubes, 1D g-C_3_N_4_ nanofibers, and g-C_3_N_4_ nanosheets, respectively. All these catalysts showed good methanol oxidation reaction (MOR) activity, but they were not tested in fuel cells. Mansor et al. [25] investigated three different graphitic carbon nitride materials as Pt supports: g-C_3_N_4_, polytriazine imide (PTI), belonging to the family of crystalline g-C_3_N_4_ materials, including Li^+^Cl^−^, and boron-doped g-C_3_N_4_ (B-g-C_3_N_4_). Before Pt deposition, following accelerated corrosion tests, all graphitic carbon nitride materials showed higher electrochemical stability than conventional carbon black, with B-g-C_3_N_4_ presenting the highest stability. Pt/PTI-Li^+^Cl^−^ showed the highest stability: the decrease in ECSA was 19% vs. 36% for Pt/Vulcan following 2000 scans.

**Figure 2:**
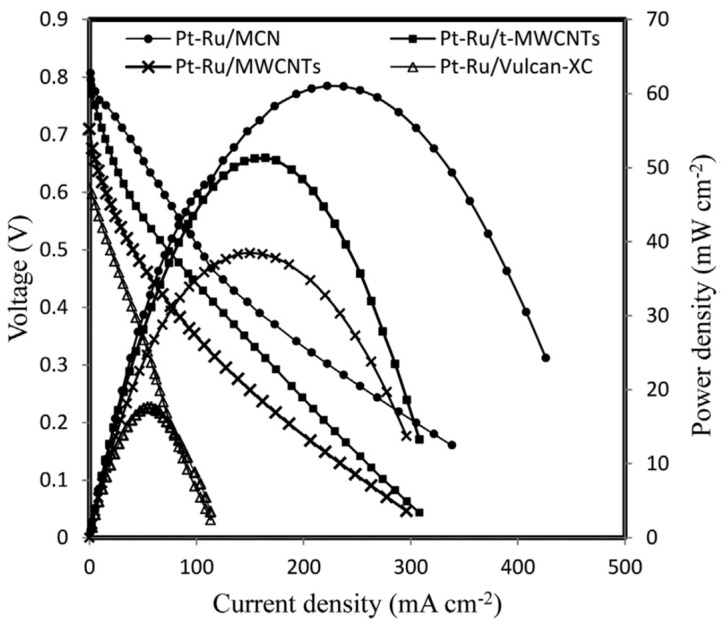
DEFC performance of Pt–Ru/MCN, Pt–Ru/t-MWCNTs, Pt–Ru/MWCNTs, and Pt–Ru/Vulcan-XC catalysts at 2 mg cm^−2^ for catalyst loading on an anode at 100 °C. Reproduced from Ref. [26], copyright 2014, with permission from Elsevier.

Goel et al. [26] compared the performance of direct ethanol fuel cells (DEFCs) with mesoporous carbon nitride (MCN), multiwalled carbon nanotubes (MWCNTs), treated t-MWCNTs, and carbon Vulcan-XC-supported PtRu anode catalysts. As shown in Figure 2, the DEFC with PtRu/MCN as the anode catalyst delivered the best performance due to the high mesoporosity and amorphous framework of the MCN support. Zhang et al. [27] fabricated a gC_3_N_4_ nanosheet-supported Pt catalyst. Pt nanoparticles were dispersed on gC_3_N_4_ nanosheets with a particle size of 1.84 nm. To increase the electron-transfer pathways on the Pt surface, acid-treated carbon black (ac-CB) was added to the Pt/gC_3_N_4_ Pt catalyst. Considerable durability of the Pt/gC_3_N_4_-ac-CB catalyst was observed. Following an accelerated durability test (ADT) of 5000 scanning cycles in a 2 mol L^−1^ H_3_PO_4_ electrolyte, the retained electrochemically active surface area (ECSA) of Pt/gC_3_N_4_–C was remarkably higher (87.6%) than that of Pt/C (54%). Moreover, during a 100 h durability test in an HT-PEMFC with Pt/C as the cathode catalyst, the voltage decay rate was 91 μV h^−1^, while a negligible decay (4 μV h^−1^) was observed for the cell with Pt/gC_3_N_4_–C as the cathode catalyst.

To overcome its poor conductivity, one solution is to add a conductive carbon material to the g-C_3_N_4_, forming a g-C_3_N_4_-carbon composite. Porous carbon black, graphene, and related materials, such as rGO, with high surface area and electrical conductivity, are commonly used as g-C_3_N_4_ substrates.

#### 2.1.2. g-C_3_N_4_-Carbon Black Composites

Qian et al. [28] prepared a Pd/g-C_3_N_4_/CB catalyst using a two-step process. First, melamine and carbon black were mixed together. Then, following heat treatment at 350 °C in an N_2_ atmosphere, the protonated melamine polymerizes formed *s*-triazine ring-based structures and bonded with oxygen-containing groups on the carbon black surface, obtaining a g-C_3_N_4_/CB composite support. The Pd/g-C_3_N_4_/CB catalyst delivered outstanding peak current densities for formic acid oxidation in acid media and methanol oxidation in alkaline media, outperforming Pd-C. This performance improvement was ascribed to the sum of three factors: the presence of CB in the composite, providing high surface area and high electrical conductivity, the g-C_3_N_4_ coating on CB, which strengthened the interaction between Pd and the support and protected the defect sites of carbon black from corrosion, and the high dispersion of Pd nanoparticles on the g-C_3_N_4_/CB composite. Lee et al. [29] prepared a core@shell support formed by a thin, layered polymeric g-C_3_N_4_ coating onto amorphous carbon black (a-CB). Pt supported on a-CB@g-CN showed enhanced ORR activity compared to a-CB-supported Pt, ascribed to a synergic effect between Pt and g-C_3_N_4_ functional groups.

**Figure 3:**
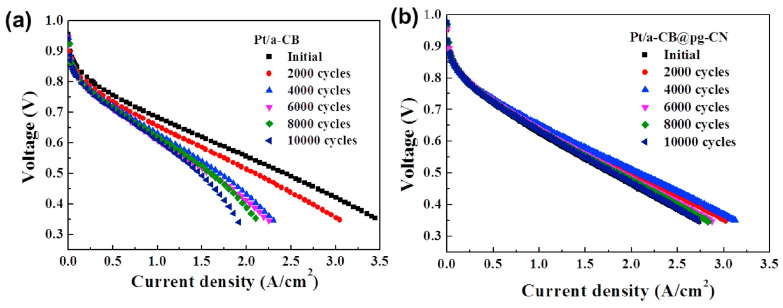
Polarization curves of PEMFCs using (**a**) Pt/a-CB and (**b**) Pt/a-CB@pg-CN cathode catalysts for a different number of potential cycles. Reproduced from Ref. [29], copyright 2018, with permission from Elsevier.

The polarization curves for different numbers of potential cycles of the PEMFCs with Pt/a-CB and Pt/a-CB@pg-CN cathode catalysts are shown in Figure 3a,b, respectively. The performance of the cell with the Pt/a-CB catalyst decreased with an increase in the number of potential cycles, while a negligible decrease in the performance was observed for the PEMFC with the Pt/a-CB@pg-CN catalyst, attesting the outstanding stability of a-CB@pg-CN support. Fang et al. [30] deposited g-C_3_N_4_ onto 3D multimodal porous carbon (MPC) (g-C_3_N_4_@MPC) and used it as an electrocatalyst support. The large hydrophobic surface area of g-C_3_N_4_@MPC results in a uniform dispersion of Pt nanowires on the support. Moreover, the 3D interconnected pore networks enhanced mass transport. For these reasons, the Pt/g-C_3_N_4_@MPC catalyst delivered improved performance compared to Pt/MPC, Pt/C, and Pt/g-C_3_N_4_@C. Yu et al. [31] prepared phosphorus-doped graphitic carbon nitride and carbon black (P-g-C_3_N_4_/CB) composite supports for Pd particles. Moreover, if the same amount of melamine (N source) (melamine) was used during the preparation process, the N content in the catalysts increased with increasing P source, indicating that the presence of P can reduce N loss during the calcination process of the mixture of melamine and CB. P doping leads to higher activity and durability for the formic acid oxidation reaction (FAOR) of Pd/ P-g-C_3_N_4_/CB than Pd/g-C_3_N_4_. Pd/ P-g-C_3_N_4_/CB with 1.5 at% P delivered a peak current density 2.15 times higher than that of Pd/C. The excellent FAOR activity of Pd/C-PCN-X catalysts was ascribed to the high dispersion of Pd particles and the change in Pd electronic structure by P doping. Alawadhi et al. [32] used a g-C_3_N_4_/VC composite material comprising of graphitic carbon nitride (g-C_3_N_4_) and Vulcan carbon (VC) as a support for Ni(OH)_2_ nanoparticles. The catalyst supported on the composite with high Vulcan carbon, i.e., 2:1, showed the best performance that is nine-times the current density compared to that over Vulcan carbon.

Liang et al. [33] synthesized g-C_3_N_4_–carbon nanosheet (CNS) composite supports with various g-C_3_N_4_ content, and then Pt/g-C_3_N_4_–CNS catalysts were prepared using the ethylene glycol reduction method. The Pt/20% g-C_3_N_4_–CNS catalyst exhibited the highest MOR activity. The addition of a certain amount of g-C_3_N_4_ to CNS anchors Pt nanoparticles, promotes the reduction of Pt^δ+^ species, and increases the specific surface area of the catalyst without changing the CNS 3D structure. The content of pyridine N and pyrrole N species increases with increasing g-C_3_N_4_ content, promoting Pt dispersion and strengthening the adhesion of Pt nanoparticles to the support, preventing agglomeration.

#### 2.1.3. g-C_3_N_4_–Graphene (Reduced Graphene Oxide) Composites

Three-dimensional hybrid g-C_3_N_4_/graphene (reduced graphene oxide) materials were investigated as fuel cell catalyst supports. Huang et al. [34] deposited homogeneous Pt particles on a hybrid 3D porous structure formed by graphene and g-C_3_N_4_, resulting in outstanding activity for methanol oxidation, high poisoning tolerance, and high stability. Li et al. [35] prepared a graphene/ultrathin g-C_3_N_4_ nanosheets composite material using a π–π stacking method and used it as a PtRu catalyst support. The electrical conductivity of g-C_3_N_4_ was promoted by the electron–hole puddle formed on the sheet interfaces between g-C_3_N_4_ and graphene. The resulting PtRu/G-g-C_3_N_4_ catalysts showed outstanding MOR activity, high poisoning tolerance, and reliable stability, ascribed to the layered structure, high surface area, ultra-fine PtRu nanoparticles, high N content, and strong metal–support interaction (SMSI). The catalyst delivered the best performance with 25% g-C3N4 in the composite support. Song and Kim [36] developed a solvothermal process for growing g-C_3_N_4_ on graphene oxide (GO) to form reduced GO (rGO) supported 3D flake-like g-C_3_N_4_ (g-C_3_N_4_@rGO) composite support. g-C_3_N_4_@rGO showed a low charge transfer resistance and a high amount of nitrogen (6–18 at.%). Pt nanoparticles with a size of 2–4 nm were uniformly dispersed on g-C_3_N_4_@rGO. In DMFCs, Pt/g-C_3_N_4_@rGO (8:1) showed higher ECSA, MOR current density, CO poisoning tolerance, and stability than Pt/rGO, Pt/C, and Pt/G. Then, Song et al. [37] prepared a polydopamine (PDA)-coated g-C_3_N_4_ on an rGO (PDA@CN-G) composite support using GO, melamine, and dopamine by using an aqueous solution process. This support has a high ECSA, various porous structures, and high N content. When Pt was supported on this composite, the Pt/PDA@CN-G catalyst showed an enhancement in MOR activity (1.5 times), high CO tolerance (3.1 times), and long-term durability (1.7 times) compared to Pt/rGO, mainly ascribed to the porous nanostructure and the synergic effect between the g-C_3_N_4_ layer and PDA coating.

**Figure 4:**
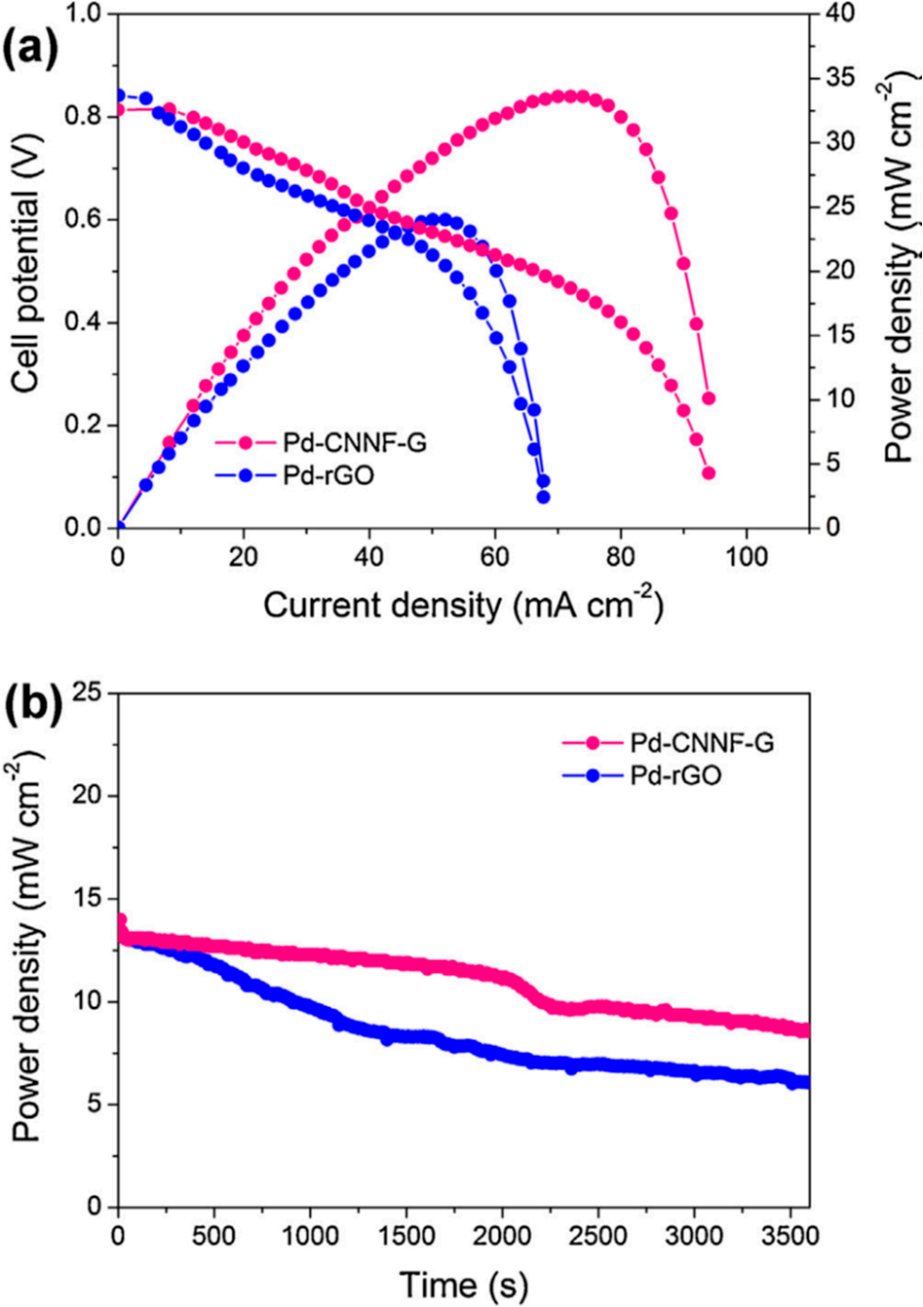
(**a**) Steady-state polarization power-density curves; (**b**) discharge curves at 0.35 V of the DFFCs using Pd-CNNF-rGO and Pd-rGO as anodes at 60 °C. Reproduced from Ref. [39], copyright 2016, with permission from Elsevier.

Zhang et al. [38] prepared covalently coupled g-C_3_N_4_ on rGO composites using in situ chemical synthesis and used them as a support for Pd nanoparticles. The Pd/g-C_3_N_4_–rGO catalyst with 9.2 wt% g-C_3_N_4_ showed a high current density for the FAOR in acid solution and for the MOR in alkaline solution, large ECSA, and reliable durability. The improved electrochemical properties of Pd/g-C_3_N_4_–rGO with 9.2 wt% g-C_3_N_4_ with respect to Pd/rGO and Pd/AC were ascribed to the concomitant effect of the high rGO conductivity and highly dispersed Pd nanoparticles on g-C_3_N_4_ planar groups. Then, Zhang et al. [39] fabricated a novel support material consisting of g-C_3_N_4_ nanoflakelets (CNNF) and rGO. Structural characterizations indicated an intimate coupling between CNNF and rGO. The CNNF can supply a large number of exposed edge sites and active nitrogen species, leading to a high dispersion of catalyst nanoparticles. The Pd/CNNF-rGO catalyst showed higher formic acid and methanol oxidation activity than Pd/rGO, Pd/AC, and Pd/CNT. The polarization and power density curves of a single direct formic acid fuel cell (DFAFC) with the Pd/CNNF-rGO and Pd/rGO catalysts are shown in Figure 4a. The DFAFC with Pd/CNNF-rGO as the anode catalyst delivered an MPD ca. 1.5 times higher than that of the cell with Pd/rGO. The stability at 0.35 V of the cell with Pd/CNNF-rGO remarkably improved compared to that with Pd/rGO (Figure 4b). Fang et al. [40] fabricated a g-C_3_N_4_@rGO composite to support Pd nanoparticles by using a simple one-step electrodeposition. Pd/g-C_3_N_4_@rGO was prepared by direct cyclic voltammetric electrolysis using a mixture of rGO and Na_2_PdCl_4_ solution and g-C_3_N_4_ dispersion. With respect to other complex methods to prepare supported catalysts containing both g-C_3_N_4_ and metal particles, electrodeposition is a one-step direct technique without heating or binding. A remarkable hybridization of g-C_3_N_4_ on rGO sheet layers was obtained, which supports the Pd nanoparticles, forming a sandwich structure on both sides of the nanosheets. Pd/g-C_3_N_4_@rGO showed outstanding MOR activity and long-term stability, considerably higher than Pd/rGO. This result was ascribed to rGO, providing high conductivity to the support and to the high N content in g-C_3_N_4_, leading to an increase in the number of anchor sites for Pd particles.

Boron was introduced in g-C_3_N_4_/G supports by doping either graphene (g-C_3_N_4_/BG) [41] or g-C_3_N_4_ (Bg-C_3_N_4_/G) [42]. The metal catalyst nanoparticles supported on these composites are effectively stabilized through their strong interactions with B and N, acting as metal trapping sites. In Pd/BG-g-C_3_N_4_, the stabilization of Pd particles on BG-CN and the strong anchoring effect of this support reduced Pd detachment [41]. The mass and specific activities of Pd/g-C_3_N_4_/BG for formic acid oxidation were 2.8- and 2.5-fold higher than those of Pd/C, respectively. This enhanced activity was ascribed to the electronic effect of the g-C_3_N_4_/BG substrate. On the other hand, the efficient immobilization of ultra-small Pt nanocrystals on 3D interweaving porous B-doped g-C_3_N_4_ nanosheet-graphene networks (Pt/Bg-C_3_N_4_-G) resulted in a large ECSA, high mass activity and poisoning tolerance, and outstanding cycling stability towards methanol oxidation, all of which exceed those of Pt/G, Pt/Bg-C_3_N_4_, PtCNT, and Pt/C catalysts [42].

### 2.2. h-BN and h-BN Nanosheets (h-BNNSs)

h-BN, with outstanding chemical stability in strong oxidative and acidic/alkaline conditions and excellent thermal stability, should serve as a stable catalyst support. Electron-rich N atoms and electron-deficient B atoms make the electron donation-back donation process easy, supplying a strong anchoring for the catalyst particle and, at the same time, boosting activity for oxygen reduction [43]. To improve electrical conductivity, vacancies and defects were introduced into the h-BN monolayer, reducing the band gap [44,45]. The presence of B and N vacancies also improves the CO tolerance of h-BN-supported Pt. Zhu et al. [46] ascribed the high CO tolerance of Pt supported on vacancy-rich BM nanosheets (h-BNNS) to an interfacial electronic effect between Pt and h-BNNS. Pt nanoparticles were embedded on both N-vacancies and B-vacancies, and the overall charge transfer was from h-BNNS to Pt, resulting in stronger O_2_ binding than CO molecules, reducing the CO poisoning of Pt. Analogously, theoretical calculations and experimental measurements indicated that the downshift of the Pd d-band center, due to the strong interaction between h-BNNSs and Pd, facilitates the removal of the reaction intermediates [47]. Recently, the suitability of h-BN as a fuel cell catalyst support was investigated [43,47,48,49,50,51]. h-BNNSs were used as supports for Pt [43,48], Pd [47,48,49,50,51], and PtPd [48] catalysts.

Three-dimensional fluffy Pt, Pd, and PtPd nanocorals (NCs) deposited on 2D h-BNNSs were obtained by a UV laser-excited photochemical reaction [48]. Laser-modification of h-BNNSs resulted in the formation of electron–hole pairs on the surfaces, improving conductivity. The PtPdNCs/h-BNNSs catalyst with a unique nanoporous surface provided high MOR activity.

**Figure 5:**
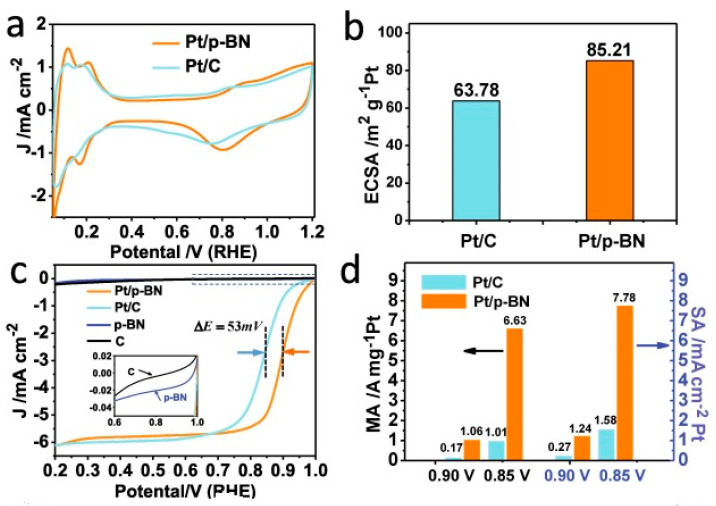
(**a**) CV curves of Pt/p-BN and Pt/C in a N_2_-saturated 0.1 M HClO_4_ solution at a 50 mV s^−1^ scan rate; (**b**) ECSA values; (**c**) LSV curves in O_2_-saturated 0.1 M HClO_4_ at a scan rate of 10 mV s^−1^ and a rotating speed of 1600 rpm; (**d**) mass and specific activities of Pt/p-BN and Pt/C at 0.85 and 0.90 V reproduced from Ref. [43], copyright 2020, with permission from Elsevier.

Porous BN (p-BN) is a type of h-BN material that has a large surface area and a high number of pores. The high porosity allows catalyst nanoparticles to anchor to the support surface. Li et al. [43] prepared a p-BN-supported Pt catalyst by using an impregnation reduction method. Ultra-fine platinum particles were dispersed on p-BN pore edges. According to the cyclic voltammetry (CV) curves in Figure 5a, the ECSA values of p-BN and Pt/C were calculated. As shown in Figure 5b, the ECSA of Pt/p-BN was larger than that of the Pt/C catalyst, attributed to the ultra-fine size and high dispersion of platinum on the substrate. ORR polarization curves for Pt/p-BN and Pt/C catalysts are shown in Figure 5c. The onset potential (*E*_onset_) and half-wave (*E*_1/2_) for the Pt/p-BN catalyst were more positive than those for Pt/C, confirming the synergic effect between Pt and p-BN. The mass activity (MA) and specific activity (SA) of Pt/p-BN and Pt/C are shown in Figure 5d. At 0.90 and 0.85 V, Pt/p-BN showed an MA of 1.06 A mg^−1^ Pt and 6.63 A mg^−1^ Pt, respectively, which are 6.23 and 6.56 times larger than those of Pt/C. The SA_Pt/p-BN_/SA_Pt/C_ ratio at 0.90 and 0.85 V is 4.59 and 4.92, which is much higher than that of ECSA_Pt/p-BN_/ECSA_Pt/C_ (1.33), indicating that the enhancement of MA of Pt/p-BN is, overall, due to the co-catalytic effect of p-BN support. Moreover, Pt/h-BN showed excellent durability in acid media. Following ADT (10,000 cycles), the ECSA of Pt/p-BN decreased by only 0.62%, and the MA by 7.1% at 0.90 V. Conversely, the ECSA of Pt/C decreased by 10.7% and the MA by 57.0%. Huang’s research group [50,51] used p-BN with different morphologies, meaning BN nanofibers (BNNFs) and BN microfibers (BNMFs), as supports for Pd nanoparticles. Pd was anchored to p-BN by an impregnation reduction method. Both Pd/BNMFs and Pd/BNNFs delivered higher FAOR activity and stability than Pd/C, ascribed to the high dispersion of the Pd nanoparticles on the p-BN surface.

## 3. ORR Catalysts

The electrochemical reduction process of oxygen consists of several elementary steps combined with a variety of parallel-continuous reactions. The oxygen reduction reaction (ORR) can be divided into two categories: a “direct” four-electron pathway to generate O_2_-species (H_2_O in acidic solutions or OH^−^ in alkaline solutions) or a “series” two-electron pathway to generate hydrogen peroxide (H_2_O_2_) [52]. The “direct” four-electron oxygen reduction pathway is recognized as the favorable pathway since H_2_O_2_ reduces energy-conversion efficiency and accelerates the degradation of the proton-conducting polymer membrane in PEMFCs. Different intermediates, including oxygenated (O*), hydroxyl (OH*), and superhydroxyl (OOH*) species, could be generated during the ORR. Several possible transformations between these intermediates make the ORR a complex process. There is still no definitive conclusion because the reaction pathway depends, to a great extent, on the catalysts and environmental parameters such as solvent, temperature, and applied electrode potential. Generally, the overall ORR rate is determined by one of these three steps: (1) the first electron transfer to the adsorbed O_2_ molecule, (2) the hydration of O_2_, and (3) the final desorption of H_2_O. Moreover, several studies reported that oxygen coverage plays a critical role in ORR mechanisms [52]. High oxygen coverage results in O–O cleavage after OOH* formation (so-called associative mechanism), whereas low oxygen coverage leads to O–O cleavage before OH* formation (dissociation mechanism). The pH of the environment also plays a crucial role in the selectivity of ORR in terms of the fact that H_2_O_2_ is more stable in acidic media than in alkaline conditions. In alkaline media, the overall reaction of the four-electron pathway is [53]

O_2_ + 2H_2_O + 4e^−^ → 4OH^−^ (0.401 V vs. SHE, pH = 14) (1)

In acid media, the overall reaction of the four-electron pathway is [53]

O_2_ + 4H^+^ + 4e^−^ → 2H_2_O (1.229 V vs. SHE, pH = 0) (2)

Going from acidic electrolytes to basic ones, a decrease in the oxygen adsorption energy takes place, allowing for the use of nonprecious catalysts [54].

Pt is commonly utilized as a PEMFC cathode catalyst; however, its ORR activity and sluggish kinetics are unsatisfactory in obtaining the required efficiency [55]. Moreover, Pt and other precious metals have high costs and limited resources. Thus, many studies have been carried out to develop nonprecious cathode catalysts [56]. Compared to PEMFCs, the main advantage of alkaline fuel cells (AFCs) is that they enable the use of nonprecious catalysts due to their high stability in alkaline media [57]. Among the different nonprecious catalysts, due to their high chemical inertness and corrosion resistance, nitrides have gained growing interest. Generally, in the literature, there is no clear distinction between nitrides as catalysts and nitrides as catalyst supports because when they are used as support, a co-catalytic effect can also be present. In this section, only nitrides acting as catalysts have been taken into account.

### 3.1. g-C_3_N_4_

g-C_3_N_4_ with high nitrogen content can act as an efficient electrocatalyst for the ORR [13,58,59,60,61], having a lot of active sites related to the lone pair electrons on the N atom. Moreover, with respect to N-doped carbon materials, g-C_3_N_4_ has a higher N content and more active and ordered sites, resulting in a more effective electrocatalyst. However, g-C_3_N_4_ has a low electronic conductivity and a low specific surface area, limiting the electrochemical reactions [13,58,59,60]. Moreover, according to theoretical calculations, it was found that g-C_3_N_4_ enables two-electron ORR transfer, resulting in the block of the catalyst surface having intermediate OOH^−^ groups [13,61]. A way to overcome these drawbacks is to synthesize g-C_3_N_4_ into either an ordered mesoporous or 1D tubular framework. Kwon et al. [62] showed that directing g-C_3_N_4_ frameworks into mesoporous architectures can generate a promising ORR electrocatalyst in an acid electrolyte. Ordered mesoporous carbon nitrides (OMCNs) were prepared by using ordered mesoporous silica as a template. OMCNs showed a remarkable enhancement in ORR activity with respect to bulk g-C_3_N_4_ and OMCs. The improved ORR activity of OMCN was ascribed to both the high surface area and the high number of N groups. Moreover, OMCNs showed superior durability and methanol tolerance to Pt/C. Tahir et al. [63] synthesized g-C_3_N_4_ in the form of nanofibers and tubular 1D nanostructures. These materials with high surface area and high nitrogen content showed good wettability regarding the electrode and an enhanced number of active sites. The tubular structure showed higher ORR activity than nanofibers and was only slightly lower than Pt/C due to the presence of a higher number of active sites and a larger surface area. Tubular g-C_3_N_4_ followed two- and four-electron transfer pathways for O_2_ in alkaline media. g-C_3_N_4_ showed higher stability and methanol tolerance than Pt/C.

#### 3.1.1. g-C_3_N_4_–Carbon Composites

**Figure 6:**
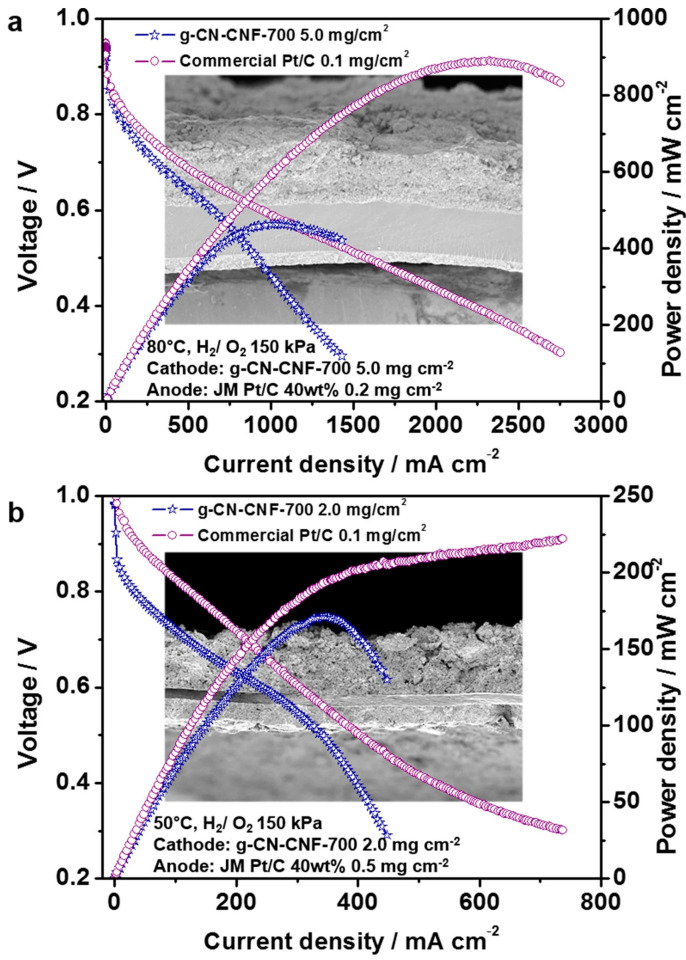
Polarization curves of PEMFCs and AFCs with g-CN-CNF-700 and Pt/C cathode catalysts. (**a**) PEMFC operated at 80 °C; (**b**) AFC operated at 50 °C. Figures in the background are cross-sectional FE-SEM images of the g-CN-CNF-based MEAs. Reprinted from Ref. [73], Kim et al. *Sci. Rep.* **2015**, *5*, 8376. https://creativecommons.org/licenses/by/4.0/ (accessed on 17 April 2025).

The problem of g-C_3_N_4_ having poor conductivity can be solved by adding a conductive carbon to the nitride. First, Lyth et al. [64] observed an improvement in the ORR activity of g-C_3_N_4_ by combining it with a high-surface-area carbon black. The conductive substrate enhances the number of electrons on the surface of g-C_3_N_4_, facilitating four-electron ORR transfer. Then, the ORR activity of different g-C_3_N_4_/carbon composites was investigated [65,66,67,68,69,70,71,72,73]. Zheng et al. [65] fabricated a graphitic carbon nitride/carbon composite via the incorporation of g-C_3_N_4_ into ordered mesoporous carbon (g-C_3_N_4_@CMK-3) using a nanocasting method. The ORR activity of g-C_3_N_4_@CMK-3 was higher than bare g-C_3_N_4_ and physically mixed g-C_3_N_4_ and CMK-3. The g-C_3_N_4_@CMK-3 catalyst showed competitive ORR activity and higher methanol tolerance when compared to Pt/C. Moreover, it showed ca. a 100% four-electron ORR transfer mechanism. Liang et al. [66] observed high ORR activity on a g-C_3_N_4_/carbon composite catalyst with 3D interconnected macropores. This composite not only showed ORR activity close to that of Pt/C but also much higher methanol tolerance and durability. Qin et al. [67] prepared a hollow mesoporous g-C_3_N_4_ nanosphere/3D graphene composite (HMCN-3G) using hydrothermal treatment of HMCN with graphene oxide. Three-dimensional graphene is a framework of interconnecting 2D graphene sheets. The HMCN-3G composite showed remarkably improved ORR activity with respect to bulk g-C_3_N_4_ and HMCN. The improvement in electrochemical performance was ascribed to the higher density of active sites, electrolyte enhancement was ascribed to the HMCN structure, and enhanced conductivity was ascribed to graphene. Fu et al. [68] synthesized 3D hierarchical porous g-C_3_N_4_/carbon composite spheres; oxygen reduction was effectively catalyzed by this composite. In addition to 3D structures, 2D graphene can also be used to improve the ORR activity of g-C_3_N_4_ due to its excellent electron-collecting and transporting properties. Yang et al. [69] prepared graphene/g-C_3_N_4_ nanosheets via graphene dispersion between g-C_3_N_4_ nanosheets using a nanocasting method. The G/g-C_3_N_4_ composite showed high conductivity along with an ORR four-electron transfer mechanism, indicating that, in addition to electronic conductivity, ORR activity increased with increasing pyridinic nitrogen content. Selvarajan et al. [70] fabricated a highly dispersed g-C_3_N_4_ matrix in graphene nanoplatelets (GNPs) via the integration of the chemical activation and polymerization of g-C_3_N_4_ precursors, resulting in a lot of active sites and enhanced interaction with the oxygen intermediates. The simultaneous effect of the g-C_3_N_4_ matrix, porosity, and conductive GNPs resulted in outstanding ORR activity, excellent stability, and methanol tolerance. Garcia et al. [71] prepared g-C_3_N_4_ supported on reduced graphene oxide (g-C_3_N_4_@rGO) using GO and urea pyrolysis. Although g-C_3_N_4_@rGO showed lower ORR activity than Pt/C, its electrochemical stability was higher. Mane et al. [72] fabricated a g-C_3_N_4_-GO composite catalyst by reducing 2D GO and porous g-C_3_N_4_ under solar radiation. This method generates Lewis base centers and a high surface area structure, making O_2_ diffusion and adsorption easier. The g-C_3_N_4_-GO electrocatalyst showed an ORR onset potential of 0.85 V, a four-electron pathway, and outstanding methanol tolerance and stability. Finally, Kim et al. [73] prepared a g-C_3_N_4_-carbon nanofiber (CNF) composite via liquid-based reactions. CNF can form secondary pores, facilitating mass transfer within the thick catalyst layer. The g-C_3_N_4_/CNF composite was pyrolyzed at 700 °C for 30 min. Figure 6 shows the performance of single cells with g-C_3_N_4_-CNF-700 and Pt/C as the cathode catalyst. For the PEMFC (Figure 6a), the current density of the cell at 0.6 V with g-C_3_N_4_-CNF-700 was 69% of the cell with Pt/C. The rapid voltage drop in the high-current-density region of the cell with g-C_3_N_4_-CNF-700 was ascribed to the much thicker electrode (50–60 μm) compared to that with Pt/C (5–10 μm), decreasing gas transport. For AFC (Figure 6b), the current density at 0.6 V of the cell with g-C_3_N_4_-CNF-700 was 80.5% of the cell with Pt/C.

#### 3.1.2. Element-Doped g-C_3_N_4_ and g-C_3_N_4_–Carbon Composites

Element doping is another promising way to improve the ORR activity of g-C_3_N_4_ [60]. Indeed, g-C_3_N_4_ doping can induce a redistribution of charges and change the effective O_2_ chemisorption mode, promoting the ORR process. Mei et al. [74] synthesized layered spongy-like oxygen-doped g-C_3_N_4_ (O-g-C_3_N_4_) by using melamine and cyanuric acid as precursors. Oxygen doping narrowed the bandgap, negatively shifted the valence band maximum, and enhanced electrical conductivity. The incorporation of oxygen resulted in a higher mass activity, a positive shift of E_1/2_ toward ORR, and a better electron transfer efficiency than pure g-C_3_N_4_. The improvement in ORR activity following oxygen doping should be ascribed to the enhancement of O_2_ adsorption and the pyridinic-N content of O-g-C_3_N_4_, which is higher than pristine g-C_3_N_4_, contributing to the formation of active sites. Xu et al. [75] synthesized 3D sulfur-doped carbon nitride (S-CN) using a melamine sponge as a precursor. CN was uniformly doped through many S–C bonds, providing additional active sites and increasing ORR activity.

**Figure 7:**
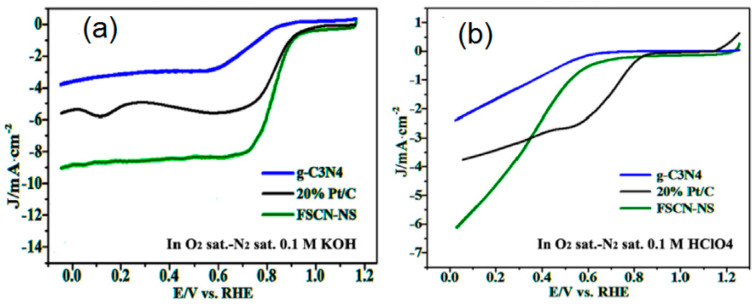
Comparison of the polarization curves of g-C_3_N_4_, FSCN-NS, and Pt/C at 1600 rpm in O_2_-saturated electrolyte at 10 mV s^−1^ in an alkaline (**a**) and acid (**b**) media. Reproduced from Ref. [77], copyright 2019, with permission from Elsevier.

S-CN showed higher ORR onset potential and electron transfer number. By using first-principle calculations and experimental measurements, He et al. [76] inferred that, among the elements of the VI A family (O, S, and Se), only O doping can remarkably improve the ORR activity of g-C_3_N_4_. Qin et al. [77] synthesized a dual core-shell g-C_3_N_4_@Fe/Sr@g-C_3_N_4_ nanosphere (FSCN-NS) catalyst by using a two-step self-assembly strategy. ORR polarization curves of g-C_3_N_4_, FSCN-NS, and Pt/C are shown in O_2_-saturated alkaline (Figure 7a) and acid (Figure 7b) media. FSCN-NS showed high ORR activity, in particular, in an alkaline medium, with an onset potential of 1.06 V. The limiting current density of FSCN-NS was higher than that of Pt/C in both conditions. The high ORR activity of FSCN-NS was ascribed to the high surface area, the high amount of pyridinic and graphitic N, and the presence of highly active ORR sites, such as Fe_3_C, FeN_x_(x = 1–3), SrCN_2_, SrC_2_, and SrFe (CN)_5_NO_2_.

The formation of element-doped g-C_3_N_4_/carbon-based material composites is a useful strategy to further improve ORR activity. An increase in the conductivity of O-g-C_3_N_4_ by 6 times was observed using Ketjen carbon black (KBC) as a support [74]. This composite showed satisfactory ORR activity, outstanding stability, and high methanol tolerance. Xu et al. [78] prepared a graphene quantum dots/sulfur-doped graphitic carbon nitride nanosheets (S-g-C_3_N_4_@GQD) nanocomposite using one-step hydrothermal treatment. This composite showed remarkably enhanced ORR activity compared to S-g-C_3_N_4_ and GQDs due to the presence of additional active sites, and it was highly effective in charge separation and the charge transfer network. Qiu et al. [79] fabricated a 3D porous phosphorus-doped graphitic carbon nitride/NH_2_-functionalized carbon black (P-*g*-C_3_N_4_@NH_2_–CB) composite using a self-assembly method. NH_2_–CB acts as a spacer, enabling self-assembly with P-*g*-C_3_N_4_ nanosheets and transforming the 2D P-*g*-C_3_N_4_ nanosheets into a 3D composite architecture with higher surface area, exposing more active sites for oxygen reduction. The outstanding ORR activity of P-*g*-C_3_N_4_@NH_2_–CB in an alkaline medium was close to that of Pt/C.

To enhance ORR activity, TMs were also used as g-C_3_N_4_ dopants. The high ORR activity of TM-doped g-C_3_N_4_ is due to the presence of TM-N-C active sites [80]. Sarkar et al. [80] synthesized copper-doped g-C_3_N_4_ (Cu-g-C_3_N_4_) using a simple one-step pyrolysis process. The Cu-g-C_3_N_4_ material showed excellent ORR activity in an alkaline medium, higher than that of g-C_3_N_4_.

**Figure 8:**
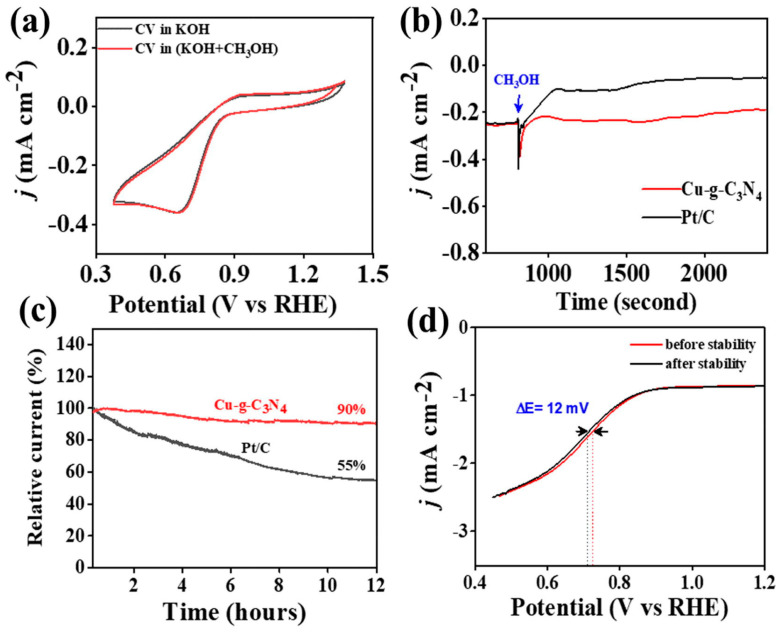
(**a**) CV curves of Cu-g-C_3_N_4_ and g-C_3_N_4_ before and after the addition of 1 M CH_3_OH; (**b**) chronoamperometry (CA) of Cu-g-C_3_N_4_ and Pt/C in O_2_-saturated 0.1 M KOH at 0.57 V with 1 M CH_3_OH added after 800 s; (**c**) CA of Cu-g-C_3_N_4_ and Pt/C at 0.57 V in O_2_-saturated KOH solution at 1600 rpm; (**d**) ORR polarization curves of Cu-g-C_3_N_4_ at 1600 rpm with 10 mV^−1^ before and after stability. Reproduced from Ref. [80], copyright 2019, with permission from Elsevier.

As can be seen in Figure 8, compared to Pt/C, Cu-g-C_3_N_4_ showed higher methanol tolerance and long-term stability, with less than 4% H_2_O_2_ formation. Then, the same research group prepared a Fe-doped g-C_3_N_4_ material using a one-step thermal polymerization reaction [81]. Fe-g-C_3_N_4_ showed outstanding ORR activity in an alkaline medium due to the presence of Fe–N_x_ active sites. This catalyst displayed an *E*_1/2_ of 0.88 V vs. RHE, following a 4e^−^ transfer pathway and outstanding stability, exceeding Pt/C. Kumar et al. [82] synthesized a CNT-supported hollow mesoporous Fe-graphitic carbon nitride (Fe-C_3_N_3_@CNTs) composite using 1,3,5-triazine polymerization with cyanuric chloride on the CNT surface. The Fe-C_3_N_3_@CNTs electrocatalysts followed the 4e^−^ ORR electron transfer pathway and showed an *E*_1/2_ that was higher than that of Pt/C. The high ORR activity of Fe-C_3_N_3_@CNTs was ascribed to the composite architecture and the high number of Fe-N_x_ sites.

Various papers have evaluated the ORR activity of composites formed by Co-doped g-C_3_N_4_ and carbon-based materials, such as CNT [83] carbon nanohorns (CNH) [84], OMC [85], graphene (G) [86] and CNT/G [87]. All these Co-containing composite catalysts showed high ORR activity, a four-electron transfer pathway, high methanol tolerance, and high stability. The improvement in ORR activity was primarily due to the presence of Co–N*_x_* active sites. Moreover, the assembly of Co-doped g-C_3_N_4_ and carbon material would lead to a highly efficient ORR process due to a strong electronic coupling between the nanostructured carbon and Co-g-C_3_N_4_. Jo et al. [88] fabricated Co- and Fe-coordinated g-C_3_N_4_ ((Co,Fe)–CN) and rGO, with the formation of many Co–N*_x_*–C and Fe–N*_x_*–C active sites. (Co,Fe)–CN/rGO showed high ORR activity, with an onset potential that was only slightly more negative than Pt/C, following a 4e^−^ ORR electron transfer pathway in acidic media. Moreover, this catalyst showed outstanding methanol tolerance and stability in acid media.

### 3.2. h-BN

Its high stability, good chemical inertness, and low price make h-BN a fascinating material for fuel cell application, but it presents intrinsic low ORR activity in the pristine form due to its low conductivity. To increase conductivity and thereby promote ORR activity, different ways, such as utilizing metal support, metal doping, carbon doping, and h-BN-carbon-based heterostructures, have been explored.

#### 3.2.1. Metal-Supported h-BN

An early work suggested that metal-supported h-BN, particularly h-BN/Ni(111), may favorably adsorb and activate O_2_ [89]. On this basis, according to a DFT analysis, Lyalin et al. [90] evaluated the ORR activity of a h-BN monolayer supported on a Ni(111) surface. They found that Ni(111) can modify the chemical and physical characteristics of defect-free monolayer h-BN, favoring the adsorption of oxygenated species. They demonstrated that inert defect-free monolayer h-BN can become ORR active via the functionalization induced by metal support; however, theoretical calculations revealed a high overpotential, limiting the ORR. Koitz et al. [91] computationally investigated the ORR of h-BN supported on Ni(111), Cu(111), and Co(111). They found that h-BN/Cu catalyzes the ORR with a low overpotential, whereas on h-BN/Ni and h-BN/Co, the presence of too-stable hydroxyl species surfaces hinders the ORR. Gao et al. [92] observed that the electron transfer from a metal substrate to h-BNNS defect sites can make h-BNNS catalytically active. They showed that the binding energies of oxygenated species on h-BNNS/Cu(111) with boron vacancies (V_B_) are similar to those on Pt(111), suggesting that inert defective h-BNNS can be functionalized by metal surfaces, becoming ORR active. Analogously, Back et al. [93] observed that noble metal supports require B (V_B_) or N (V_N_) vacancies to make h-BN active for oxygen reduction. It was observed that different kinds of h-BN on gold substrates act as ORR catalysts [94,95].

**Figure 9:**
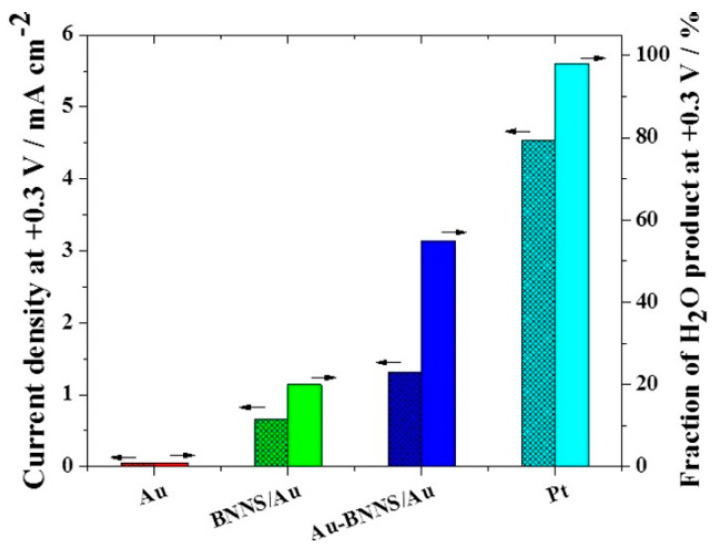
Dependence of the current density at 0.3 V and the fraction of H_2_O produced on Au, BNNS/Au, Au-BNNS/Au, and Pt electrodes. Reproduced from Ref. [96], copyright 2016, with permission from Elsevier.

Among the various h-BN forms, the highest ORR activity was achieved by h-BNNS, but the overpotential was much higher than that of a Pt electrode, and almost 100% oxygen reduction followed a two-electron transfer pathway [95]. ORR activity strongly depends on the h-BNNS-Au interactions. As shown in Figure 9, an increase in the h-BNNS-Au interaction by decorating h-BNNS/Au with Au nanoparticles leads both to higher ORR activity and to a higher amount of H_2_O produced [96,97]. When small Au nanoparticles were used for h-BNNS decoration, a very low ORR overpotential was observed (350 mV lower than at the Au electrode and only 100 mV higher than at the Pt electrode), and 80–90% oxygen reduction followed a four-electron transfer pathway. Moreover, the utilization of small-sized BNNS increases h-BNNS-Au interactions. Dinh et al. [98] studied the influence of the size of Au-supported h-BNNS on ORR activity in an acid medium. The ORR overpotential decreased with decreasing h-BNNs size due to the increase in the active sites. The modification of the Au surface with the smallest-size h-BNNS resulted in a decrease in the overpotential by ∼330 mV with respect to the unmodified Au electrode.

#### 3.2.2. Metal-Doped h-BN

The presence of defect sites in an h-BNNS, such as V_B_ and V_N_, enhances its chemical reactivity [99]. While the chemical inertness of defect-free h-BNNS results in a weak interaction with metal atoms, the defective h-BNNS is suitable for developing novel catalysts by metal doping. DFT calculations were used to evaluate the ORR activity of metal-doped defective BNNSs [100,101,102,103]. Feng et al. [100] showed that Fe atoms can strongly bind with V_B_ sites of defective h-BNNs, ensuring high stability. Based on the free energy change and activation energy of each step in the ORR process, it can be inferred that Fe-h-BNNS possesses good ORR activity through a direct four-electron pathway. Esrafili et al. [101] evaluated Si-embedded boron nitride nanotubes (Si-BNNTs) as an ORR electrocatalyst. There are two substitution sites in Si-BNNTs: a B site (Si_B_) and an N site (Si_N_). The results indicated that Si_B_-BNNT has outstanding ORR activity. Hsu et al. [102] reported that in Mn-doped h-BN, Mn atoms have a higher probability of replacing boron in h-BN vacancies than nitrogen. Moreover, Mn atoms have a high ability to adsorb oxygen-containing intermediates, ascribed to the large charge difference between N and Mn. Finally, by using DFT simulations, Chen et al. [103] investigated h-BN embedded with 23 transition metal (TM) atoms as highly active single-atom ORR electrocatalysts. Based on the screening criterion (−4.92 eV < Δ*G*_O_* < 0 eV), 13 kinds of TM-BN met the criterion and were selected to study their ORR performance. Among them, Au-BN delivered the highest ORR activity with the lowest overpotential, lower than that of Pt. Experimental measurements should be carried out to validate the theoretical results.

#### 3.2.3. Carbon-Doped h-BN

The presence of C atoms in the h-BN lattice, either as isolated dopants or as C “islands”, can significantly improve its electron mobility [104]. The C_B_ or C_N_ defect sites, formed by the replacement of B or N atoms with C atoms, can act as n-type or p-type sites or Lewis acid-base sites in electrocatalytic reactions. Theoretical calculations showed that the C_N_ sites can act as sites for oxygen reduction, balancing O_2_ adsorption and desorption. The isolated C-doped sites [105,106,107] or smaller graphene islands [108] can effectively break the O-H bond in H_2_O. Thus, C-h-BN materials, due to their outstanding activity for O_2_ reduction to H_2_O, are promising fuel cell metal-free cathode catalysts. By using DFT calculations, Zhao et al. [105] investigated the use of carbon-doped h-BNNS as an ORR catalyst. The results indicated that single C-doped h-BNNS can increase charge density and decrease the energy gap, leading to an improvement in O_2_ adsorption. The C_N_ sites are highly effective for O_2_ activation and promote the ORR steps to follow a four-electron pathway. Nguyen et al. [106] studied the ORR activity of C-doped h-BN nanoflakes by using quantum chemical calculations; both single and double C-doped systems are ORR active, but the latter, with two unpaired electrons, is more efficient than the former, with one unpaired electron. Gao et al. [107] observed that C_B_ generates an n-type semiconductor h-BN with appreciable O_2_ activation activity in a large area extended far away from the C_B_ defect. O_2_ adsorption energy on C_B_@h-BN decreases slowly with increasing distance from C_B_, keeping O_2_ highly activated. Conversely, for monolayer h-BN doped with TMs and different atoms of III, IV, and V groups, O_2_ adsorbs only close to the dopant. Marbaniang et al. [108] synthesized C-doped h-BN using a chemical vapor deposition method. The catalyst obtained after annealing at 850 °C showed remarkable ORR activity and high stability even up to 10,000 potential cycles. Finally, Zhang et al. [109] co-doped h-BN with 3d TMs and carbon atoms. The TM atoms, anchored with four or two C atoms, formed TM–C_4_–BN and TM–C_2_N_2_–BN structures. TM–C_2_N_2_–BN showed higher structural stability and stronger adsorption of oxygen-containing intermediates than TM–C_4_–BN. The best catalyst for oxygen reduction was Co–C_2_N_2_–BN.

#### 3.2.4. Graphene-h-BN Composites

Unlike h-BN, graphene has almost metallic properties; thus, combining the advantages of graphene with those of h-BN can be a proper strategy. Different studies indicated that in-plane graphene/boron nitride (G/h-BN) heterostructures can act as a proper ORR catalyst [110,111,112,113]. Patil et al. [110] synthesized an rGO/h-BN nanocomposite by using a one-step hydrothermal method followed by high-temperature annealing. The rGO/h-BN nanocomposite showed high ORR activity with a single-step, almost four-electron transfer pathway and an onset potential of ~0.8 V vs. RHE in an alkaline medium. Moreover, the rGO/h-BN nanocomposite showed higher stability after 10,000 cycles and higher methanol tolerance than Pt/C. Sun et al. [111] demonstrated that the C–N interfaces of G/h-BN heterostructures act as active sites. H_2_O formation at the C–N interface is both energetically and kinetically favored. Patil et al. [112] attributed the outstanding ORR activity of a CNT/h-BN nanocomposite to a synergic effect between CNT and h-BN. Rastogi et al. [113] prepared a vertical non-van der Waals (non-vdW) G/h-BN heterostructure. This non-vdW G/h-BN, unlike vdW heterostructures, forms a chemical bridge between graphene and h-BN, leading to a direct charge transfer and high ORR activity, similar to that of Pt/C, but with higher stability and methanol tolerance.

#### 3.2.5. Tri-Functional h-BN-Based Electrocatalysts

**Figure 10:**
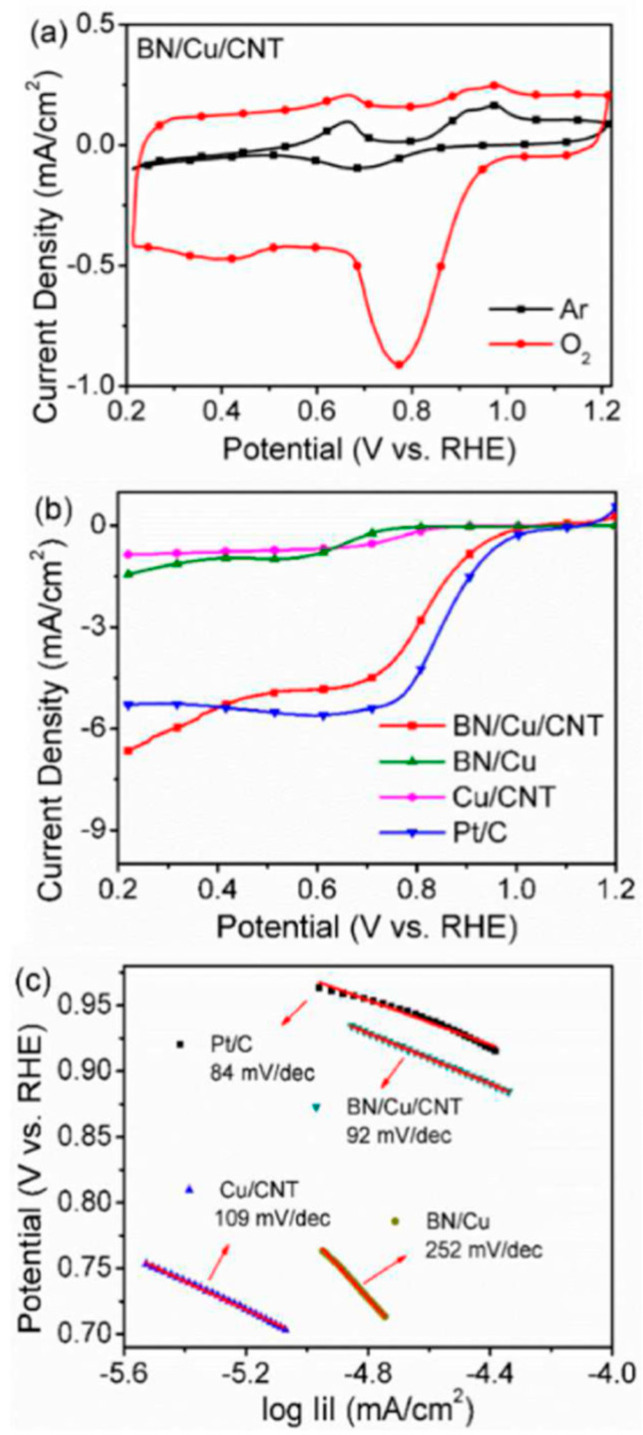
(**a**) CV curves of h-BN/Cu/CNT in O_2_-saturated and Ar-saturated 0.1 M KOH. (**b**) LSV curves and (**c**) Tafel plots of h-BN/Cu/CNT, h-BN/Cu, Cu/CNT, and Pt/C catalysts in O_2_-saturated 0.1 M KOH. Reproduced from Ref. [115], copyright 2023, with permission from Elsevier.

As previously reported, the ORR activity of h-BN can be improved in the presence of a metal. The poor electronic conductivity of h-BN, however, limits the use of h-BN/metal in fuel cells. By combining it with graphene or CNTs, the electrical conductivity of h-BN/metal can be greatly improved. On this basis, the ORR activity of tri-functional electrocatalysts was investigated. Chen et al. [114] investigated the ORR activity of tri-functional h-BN/TM/G catalysts using DFT calculations. A series of 3d and 4d TM atoms covered by h-BNNS and graphene were screened in detail. A volcano relationship between ORR overpotential and ∗OH binding strength (Δ*G*_∗OH_) was established, revealing that when the Δ*G*_∗OH_ is in the range of 0.5–1.2 eV, the BN/TM/G catalysts will present relatively high activity for oxygen reduction. For Co, Mo, Ru, Tc, Mn, Cu, Ni, and Fe metals located near the top of the volcano curve, their corresponding h-BN/TM/G catalysts present a relatively small ORR overpotential. Among them, h-BN/Cu/G showed the lowest ORR overpotential. Yu et al. [115] prepared a tri-functional h-BN/Cu/CNT catalyst by using a complexation-reduction method. This electrocatalyst showed high ORR activity, delivering a high cathodic current density (Figure 10a) comparable to that of Pt/C. The ORR linear sweep voltammetry (LSV) curves of h-BN/Cu, Cu/CNT, h-BN/Cu/CNT, and Pt/C catalysts are shown in Figure 10b. The ORR onset potential of h-BN/Cu/CNT was more positive than that of h-BN/Cu and Cu/CNT, and it was close to that of Pt/C. Unlike h-BN and CNT alone, h-BN and CNT together strongly enhance the ORR activity of Cu nanoparticles. The Tafel plots of h-BN/Cu, Cu/CNT, and h-BN/Cu/CNT were 252, 109, and 92 mV dec^−1^, respectively, while that of Pt/C was 84 mV dec^−1^ (Figure 10c), which is in agreement with LSV results.

## 4. Composite Membrane

Perfluorosulfonic acid (PFSA) membranes, such as Nafion, are the commonly utilized PEMs in low-temperature fuel cells due to their excellent chemical, mechanical, and thermal stability, as well as high proton conductivity under hydrated conditions [116]. However, some limitations of PFSA membranes, such as poor proton conductivity in anhydrous conditions, high manufacturing costs, high fuel permeability, low chemical stability, and the degradation of their properties at high temperatures, have been addressed in the research into alternative PFSA PEMs, such as sulfonated poly (ether ketone ether) (SPEEK), polybenzimidazole (PBI), and sulfonated poly(ether sulfone) (SPES) PEMs. These membranes, however, suffer from poor proton conductivity and mechanical stability. A way to overcome these drawbacks is the addition of various multifunctional organic, inorganic, and hybrid fillers [117]. Composite membranes with h-BN and g-C3N4 fillers are a promising solution.

### 4.1. h-BN and h-BN-Containing Composite Membranes

#### 4.1.1. h-BN Membranes

h-BN has great potential as a PEMFC membrane, as it has high proton conductivity and outstanding gas-blocking capability [117]. First, single-layer h-BN (1L-BN) and an AA’-stacked tri-layer h-BN (3L-BN) were tested as fuel cell PEMs. The PEMFC with a 3L-BN membrane delivered a better performance than that with the 1L-BN membrane [118]. The change in the open circuit voltage (OCV) and power density of the cells with 1L-BN and 3L-BN membranes following 100 h of accelerated stress tests (ASTs) was of little importance, whereas the OCV and the power density of the cell with a Nafion 211 membrane remarkably decreased. However, structural defects and mechanical damage during the transfer of the h-BN layer and membrane swelling, as well as hydrophobicity and low surface charge, limited the application of h-BN to PEMFCs. To enhance the surface charge and stability, Jia et al. [119] fabricated Nafion-functionalized h-BN-based membranes (NBN). This membrane facilitates proton conduction within the membrane. At appropriate Nafion contents, the NBN membranes showed high proton conductivity and good stability in a fuel cell environment.

#### 4.1.2. h-BN-Nafion Composite Membranes

Different works addressed h-BN-filled Nafion, aiming to improve proton conductivity, particularly in anhydrous conditions, and reduce fuel crossover [120,121,122,123]. Jia et al. [120] blended NBN with Nafion to obtain NBN/Nafion composite PEMs. The addition of NBN to Nafion provided more proton transportation sites and improved water uptake. The proton conductivity of the composite membrane remarkably increased compared to recast Nafion. At 80 °C–95% RH, the proton conductivity of 0.5 NBN/Nafion was 6 times that of recast Nafion. Akel et al. [121] fabricated composite membranes by mixing Nafion and 3, 5, 10, and 15 wt% h-BN nanoparticles. The presence of hydrophobic h-BN particles decreased the swelling property and methanol retention of Nafion. In dry conditions, the maximum proton conductivity of the membrane was obtained with 10 wt% h-BN; this was much higher than pure Nafion. The enhanced proton conductivity was ascribed to H-bond formation between the amine and hydroxyl groups of h-BN and the sulfonic acid groups of Nafion. Parthiban and Sahu [122] evaluated sulfonated h-BN as a potential filler to prepare Nafion composite membranes. h-BN layered structure morphology efficiently reduced methanol crossover, while SO_3_^−^ presence enhances proton conductivity. The proton conductivity of Nafion–h-BN in the optimum composition was ∼58% higher than that of Nafion. The single-cell DMFC with a Nafion–h-BN (0.75 wt%) membrane delivered an MPD of 165 mW cm^−2^ at 70 °C, while the cell with a pristine Nafion membrane showed an MPD of only 65 mW cm^−2^. Similarly, Lee et al. [123] coated an ultra-thin h-BN layer on a Nafion membrane. The h-BN-coated layer efficiently suppressed the gas crossover, hindering oxygen radical formation in the electrodes without decreasing Nafion proton conductivity.

#### 4.1.3. h-BN-Non-Perfluorinated Polymer Composite Membranes

Among different PEMs, SPEEK is one of the most promising PFSA-alternative membranes due to its outstanding chemical and thermal stability, excellent strength, low fuel crossover, and low cost [124]. The control of the degree of sulfonation (DS), on which the proton conductivity of SPEEK depends, is essential. A high DS will produce high proton conductivity, but excessive DS will have negative effects on membrane stability. Meanwhile, a low DS will result in not enough proton conductivity. Thus, an optimal DS of SPEEK is very important to balance its effects, but this is not simple. For this reason, other ways to increase SPEEK proton conductivity and mechanical stability were explored, such as blending SPEEK with organic and inorganic fillers. Oh et al. [125] investigated boron nitride nanoflakes (BNNFs) as a SPEEK nanofiller. A low content of BNNFs (0.3 wt%) remarkably improved the mechanical stability of SPEEK during repeated wet/dry cycles. As it is difficult to disperse BNNFs in most organic solvents, it was non-covalently functionalized with 1-pyrenesulfonic acid (PSA). In addition to a high dispersion, PSA sulfonic functional groups improved proton conductivity. The SPEEK/BNNFs composite membrane showed considerably improved long-term durability compared to pristine SPEEK. Huang et al. [126] prepared an ultra-thin reinforced membrane by using h-BNNSs, high sulfonated SPEEK, and expanded polytetrafluoroethylene (ePTFE). The MPD of the PEMFC with the 0.5%-BN@SPEEK/PTFE membrane was higher (by 16.6%) than that of the cells with the SPEEK/PTFE and Nafion 212 membranes. Yadav et al. [127] added sulfonated boron nitride (SBN) to a SPEEK matrix for its use in DMFCs. The presence of SBN reduced methanol crossover and increased the ion exchange capacity (IEC), proton conductivity, and mechanical strength of the membrane. Yogarathinam et al. [128] used polyaniline-coated activated boron nitride (PANI-A-BN) as a filler in SPEEK membranes. In the presence of PANI-A-BN, methanol permeability was significantly reduced, and proton conductivity and chemical stability were improved. The power density of the DMFC with the PANI-A-BN/SPEEK membrane was higher than that with the pristine SPEEK membrane.

SPES and PBI are other non-fluorinated polymers widely used in fuel cells; however, their proton conductivity and stability are not satisfactory. Functionalized h-BN was used as a filler for SPES [129,130]. Galhot et al. [129] functionalized h-BN using acid treatment, inducing h-BN exfoliation as well as sulfur functionalization. The incorporation of a low amount of functionalized h-BN (0.5 wt%) into the SPES enhanced the mechanical and thermal stability and proton conductivity of the membrane. The filler acts as a barrier for methanol and facilitates proton transport. Kumar et al. [130] prepared hydroxylated boron nitride (HBN) using liquid exfoliation and the hydroxylation of h-BN, yielding few-layered sheets. An outstanding synergic effect between SPES and HBN through the functional groups (SO_3_H–OH) occurred, resulting in continuous proton transfer channels. The power density of the PEMFC with a 3.5 wt% HBN/SPES membrane was improved compared to that with pristine SPES, with reduced membrane degradation after a 120 h durability test. Hussin et al. [131] prepared PBI/h-BN composite membranes, with the h-BN loading between 2.5 and 10 wt%. The HT-PEMFC with PBI/BN-2.5 membrane delivered better performance than that with a pristine PBI membrane.

**Figure 11:**
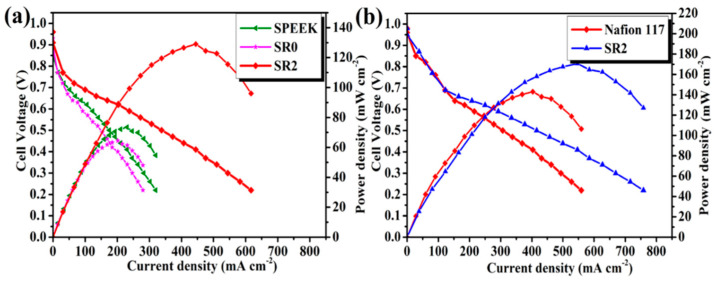
Polarization and power density curves of PEMFCs with (**a**) SPEEK, SR0, and SR2 membranes at 50 RH% and (**b**) SR2 and Nafion117 membranes at 75 RH%. Reproduced from Ref. [132], copyright 2022, with permission from Elsevier.

The effect of functionalized h-BN addition to composite SPEEK/P (P = PES, PBI) membranes on their properties was also evaluated [132,133]. Polydopamine-modified boron nitride (AH-BN) sheets containing base groups (–NH_2_ and –NH–) were synthesized and incorporated into a SPEEK/PES composite membrane [132]. Acid–base pairs were formed at the SPEEK-PES/AH-BN interface, offering continuous pathway channels for proton transport via a Grotthuss mechanism. The performance of the PEMFCs with SPEEK, SPEEK/PES (SR0), and SPEEK/PES/AH-BN (3 wt%, SR2) membranes at 80 °C at 50 RH% is reported in Figure 11a. The performance of the cell with the SR2 composite membrane was the highest. The PES and AH-BN sheets and the 2D structure remarkably reduce the fuel blockage of the hydrogen crossover across the composite membrane. The performance of the cell with the SR2 composite membrane was evaluated at 80 °C and 75 RH% and compared with that of the cell with a Nafion 117 membrane. The obtained OCV was nearer to that of the PEMFC with a Nafion 117 membrane (Figure 11b), and the MPD value was higher in comparison. Harameen et al. [133] functionalized h-BN by introducing a hydroxyl group and then added it to SPEEK/PBI. The addition of h-BN remarkably improved the protonic conductivity of the composite membrane, and a noticeable enhancement in thermal stability and ionic conductivity was observed.

### 4.2. g-C_3_N_4_-Containing Composite Membranes

Two-dimensional ultra-thin g-C_3_N_4_ has been utilized as filler in polymer membranes due to its unique properties [13]. The amino (–NH_2_) and imino (–NH) groups of g-C_3_N_4_ are expected to interact with the sulfonate groups (−SO_3_^−^) in the polymer matrix, forming acid–base pairs, promoting −SO_3_^−^ dissociation and improving Grotthuss-type proton transfer [13]. The incorporation of g-C_3_N_4_ significantly improves proton conductivity, thermal stability, and the strength of the membrane. Unlike h-BN, which is utilized as a filler only in proton exchange membranes, g-C_3_N_4_ was used in both proton and anion exchange membranes. –NH_4_ and OH^−^ groups in g-C_3_N_4_ can form H-bonds with OH^−^ and H_2_O, promoting the dissociation of quaternary ammonium groups in the anionic membranes [134]. g-C_3_N_4_ was added to protonic SPEEK [135,136,137,138,139,140] and sulfonated poly (arylene ether sulfone) (SPAES) [141,142,143,144] and anionic quaternary poly (arylene ether ketone) (QPAEK) [134,145] membranes. To enhance the proton conductivity and stability of polymer membranes, mesoporous g-C_3_N_4_ [141], F- [146], O- [138], P- [139], and H-doped [140] g-C_3_N_4_ and sulfonic acid-functionalized [147] g-C_3_N_4_ were used as fillers. To further improve membrane performance, the utilization of a double filler instead of a single filler was also reported, combining not only the properties of different materials but also exploiting a synergic effect between them. The addition of double-filler composites, formed by adding g-C_3_N_4_ and either an oxide compound (GO, CeO_2_, TiO_2_, and Gd_2_Zr_2_O_7_) [142,143,148,149,150] or phosphotungstic acid (H_3_PW_12_O_40_; HPW) [135,139,141,144] to a polymer membrane, was reported with promising results.

Graphitic carbon nitride–gadolinium zirconium oxide (g-C_3_N_4_–Gd_2_Zr_2_O_7_) is a promising double filler that can simultaneously increase the power density and lifetime of polymer membranes in high-temperature PEMFCs.

At 100 °C under 30% RH, the MPD of the cell with a Nafion/gC_3_N_4_–Gd_2_Zr_2_O_7_ membrane was 504 mW cm^−2^ at a current density of 804 mA cm^−2^, which is 1.9- and 2.3-fold higher than that of the cell with Nafion-212 and pristine Nafion membranes. Moreover, the cell with Nafion/gC_3_N_4_–Gd_2_Zr_2_O_7_ displays an OCV decay of 0.1 mV h^−1^ during 515 h of operation, which is remarkably lower than that of the cell with a Nafion membrane during 300 h of operation (2.8 mV h^−1^) [149]. At 125 °C under 15% RH, the PEMFC with the Nafion/gC_3_N_4_–Gd_2_Zr_2_O_7_ membrane largely outperforms the cells with Nafion-212 and pristine Nafion membranes, delivering a power density of 380 mW cm^−2^ [149]. Vinothkannan et al. [150] used a SPEEK/gC_3_N_4_–Gd_2_Zr_2_O_7_ composite membrane in an HT-PEMFC, attaining an MPD of 315 mW cm^−2^ at 110 °C under 15% RH, with minimal chemical degradation after 300 h of operation.

HPW, as a solid heteropolyacid, has strong acidity, hydrophilicity, outstanding stability, and high proton conductivity.

**Figure 12:**
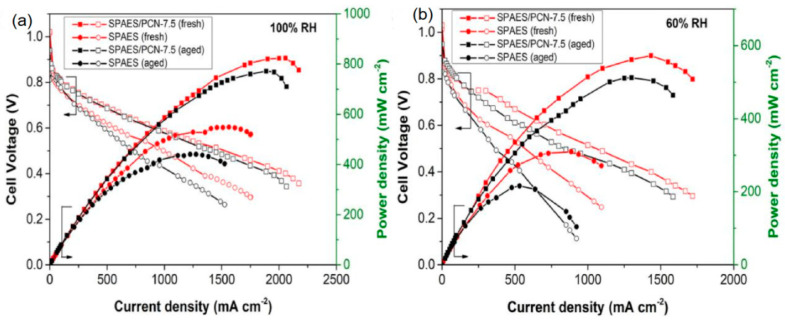
Polarization and power density curves of PEMFCs with SPAES and SPAES/PCN-7.5 membranes at 80 °C under (**a**) 100 RH% and (**b**) 60 RH% before and after the durability test. Reproduced from Ref. [141], copyright 2024, with permission from Elsevier.

HPW can develop a preferential pathway for proton transport, enhancing water retention and decreasing the energy barrier of proton conduction. However, HPW is highly soluble in water; to prevent its leakage from the membrane, one way is to immobilize it onto g-C_3_N_4_ nanosheet surfaces. The immobilization of HPW onto g-C_3_N_4_ nanosheet surfaces not only combines the advantages of HPW and 2D g-C_3_N_4_ nanosheets but also can effectively inhibit the leakage of HPW. Figure 12a,b show the polarization curves of the PEMFC with 7.5 wt% of HPWg-C_3_N_4_/SPAES (SPAES/PCN-7.5) and bare SPAES membranes under 60/100 RH%s at 80 °C before and after a durability test. Before the stability test, the SPAES/PCN-7.5 membrane displays a remarkably better PEMFC performance at both RH% conditions than pristine SPAES. The cell with the SPAES/PCN-7.5 membrane attained MPD values 1.5 (at 100 RH%) and 1.95 (at 60 RH%) times, respectively, that of the cell with the SPAES membrane due to the excellent proton conductivity of the composite membrane. After the durability test, the MPD values of the PEMFC with the SPAES membrane decreased by 30.4 % and 19.4 % at 60 and 100 RH%, respectively. The cell with the SPAES/PCN-7.5 membrane, instead, showed a decrease in MPD of 10.5 % and 6.3 %, indicating the remarkable long-term stability of the composite membrane formed by HPW/g-C_3_N_4_ nanosheets and SPAES.

The addition of g-C_3_N_4_ enhances the proton conductivity of different membranes by 28–127%, and, as a consequence, fuel cell performance is also enhanced by 17–164%. Among the different composite membranes, the incorporation of CQD/g-C_3_N_4_ in the SPEEK membrane resulted in the highest enhancement in proton conductivity and MPD [135].

## 5. Summary and Future Prospects

The advantages and disadvantages of the use of pristine, doped, and composite g-C_3_N_4_ and h-BN in low-temperature fuel cells are highlighted in Table 1.

Catalyst supports. To overcome the shortcomings related to the use of carbon, different types of alternative supports have been evaluated. Among them, its lower price, excellent mechanical resistance, and outstanding chemical stability under high temperatures and severe conditions make g-C_3_N_4_ suitable for use as a fuel cell catalyst support. N atom presence provides a proper synergic bonding environment to anchor catalyst particles. Triazine units can form an effective coordination complex with catalyst particles, enhancing electrocatalyst stability. A hindrance to the utilization of g-C_3_N_4_ as a fuel cell catalyst support is its poor conductivity and low surface area. A way to increase the conductivity is the addition (to g-C_3_N_4_) of a conductive carbon material. Compared to the catalysts supported on commonly used carbon, catalysts supported on nanostructured g-C_3_N_4_ and g-C_3_N_4_/carbon composites showed high catalytic activity and stability. The fuel cells with the nanostructured g-C_3_N_4_ and hybrid g-C_3_N_4_/carbon-supported catalysts delivered better performance than the cells with carbon-supported ones. Meanwhile, h-BN has emerged as a new catalyst support material, showing promising performance and durability. Porous h-BN and h-BN nanosheets were investigated as fuel cell catalyst supports using out-of-cell tests. The catalysts supported in these materials showed higher catalytic activity and stability than the same catalysts supported on carbon. In the case of h-BN, unlike g-C_3_N_4_, to increase surface area and electrical conductivity, it is preferable to use porous and defective structures rather than nitride/carbon composites.

A key factor in the preparation of a suitable catalyst support is the design of the porous structure of the electrode composed of the catalyst and catalyst support materials. Research efforts have to address the synthesis of exfoliated 2D layered nitride structures or also holey structures to tune pore size and to form composites with other catalyst support materials naturally possessing the required porosity.

Catalysts. Compared to other N-carbon materials, g-C_3_N_4_ has higher N content and more active sites, resulting in a more effective metal-free ORR electrocatalyst. Compared with Pt catalysts, g-C_3_N_4_ has numerous advantages, such as lower cost and greater abundance, higher methanol poisoning tolerance, and the possibility of creating various nanostructures by using a templating method. However, g-C_3_N_4_ has low electronic conductivity and low specific surface area, limiting electrochemical reactions. The poor conductivity of g-C_3_N_4_ can be overcome by using a conductive carbon support. Element doping is another promising way to improve the ORR activity of graphitic carbon nitride. Its high stability, good chemical inertness, and low price make h-BN a fascinating material for fuel cell application, but it suffers from intrinsic low ORR activity in the bare form, owing to its low conductivity. To increase conductivity, different strategies, such as using a metal support, metal doping, carbon doping, and h-BN-carbon-based material heterostructures, have been reported. Regarding metal-doped h-BN, only theoretical calculations have been reported. Experimental measurements should be carried out to validate the theoretical results.

Doped and/or composite g-C_3_N_4_ and h-BN catalysts showed high methanol tolerance, high stability, and an ORR close to and, in some cases (in particular, in alkaline media), higher than that of Pt. It is important to note that, unlike other uses, most experiments regarding the use of nitrides, in particular h-BNs, as catalysts, have been carried out using out-of-cell tests. More tests in fuel cells should be performed to better evaluate these materials. This is a key point. Indeed, very often, even if a low ECSA loss was obtained following durability tests in a half-cell, indicating good durability, fuel cell performance dropped over time due to poor water and heat management in the membrane-electrode assembly. Thus, more studies should address the long-term durability of g-C_3_N_4_ and h-BN catalysts in a single fuel cell test to evaluate the suitability of electrodes composed of these catalyst systems in an actual fuel cell environment. Moreover, the degradation mechanisms of g-C_3_N_4_ and h-BN catalysts should be deeply investigated.

An issue in the preparation of nitride catalysts is balancing the micro- and mesopores where the active sites are located. This is a key factor in enabling the accessibility of the reactants and preventing water from pooling at the pores. This is considered an important challenge in fabricating g-C_3_N_4_ and h-BN-based MEAs, requiring a high active surface area for ORR activity (presence of micropores) and good mass transport (presence of mesopores) during the reactions.

To improve the electrocatalytic properties of g-C_3_N_4_, it is suggested that g-C3N4 structures be fabricated with a continuous phase of single atomic thickness. It can be obtained by optimizing the reaction conditions, like temperature, and selecting a suitable precursor in the gaseous phase. Moreover, post-treatments like exfoliation and etching (during hard template synthesis) can introduce defects that are useful for improving ORR activity.

Exfoliation is an important strategy in the preparation of h-BN nanosheets used as fuel cell catalysts. However, some challenges exist in the process of exfoliation. Commonly, 2D h-BN nanosheets are exfoliated from bulk h-BN through long-duration ultrasonic treatment in a polar solvent, mechanical exfoliation, ion insertion, and ball milling. However, the process mentioned above is too complicated and/or hard to scale up for real applications. Because of the lower yield, low stability and the restacking of layers are observed in exfoliated layers. Solvothermal techniques are widely used to exfoliate many two-dimensional materials, but the impurities are prone to attaching to the BNNSs exfoliated by the solvothermal route [151]. Thus, research efforts have to address obtaining g-C_3_N_4_ and h-BN nanosheets derived from the bulk materials using improved methods, such as covalent, non-covalent, and Lewis acid–base approaches.

Finally, future studies should focus on the knowledge of the basic mechanisms associated with the catalytic process. Such attempts will help in the development of more suitable g-C_3_N_4_ and h-BN catalysts.

As previously reported, nitride/carbon composite materials have been proposed as catalyst supports and ORR catalysts to improve nitride properties. However, considering the unsatisfactory stability of carbon-based materials in a fuel cell environment, the use of nitride/carbon composites to improve surface area and electrical conductivity is not appropriate. Thus, research efforts should address the development of novel carbon-free nitride-based materials, such as nanostructured nitrides, metal-doped nitrides, and also nitride/ceramic oxide composites, such as triple metal oxide (CoNiMn)/porous g-C_3_N_4_ composites, showing encouraging results as ORR catalyst [152].

When g-C_3_N_4_ is used as a catalyst support, one way to improve conductivity is to develop new synthesis methods. For example, Shi et al. [153] proposed an innovative gamma irradiation method to efficiently synthesize g-C_3_N_4_-supported Pt. Using this method, conductive channels between the g-C_3_N_4_ and supported Pt are formed. The Pt/g-C_3_N_4_ was tested for ORR and showed a small Tafel slope and a four-electron transfer path. The ORR activity of Pt/g-C_3_N_4_ was much higher than that of Pt/C. Another way to improve the conductivity of g-C_3_N_4_ without the addition of a carbon material is the addition of Ag [154]. In addition to enhancing electrical conductivity, the presence of Ag improves the CO tolerance of g-C_3_N_4_.

Membrane fillers. To overcome the drawbacks related to the use of PFSA membranes, non-fluorinate membranes, such as SPEEK and PBI, have been proposed. However, they suffer from poor proton conductivity and mechanical stability. The addition of stable and proton-conducting g-C_3_N_4_ and h-BN to these membranes is an effective way to improve their characteristics. Unlike h-BN, which is used as a filler only in proton exchange membranes, g-C_3_N_4_ was used in both proton and anion exchange membranes. Amino and hydroxyl groups in g-C_3_N_4_ can form hydrogen bonds with OH^−^ and water, promoting the dissociation of quaternary ammonium groups in the anionic membranes. The addition of g-C_3_N_4_ enhances the proton conductivity of different membranes by 28–127%, and, as a consequence, fuel cell performance is also enhanced by 17–164%. The utilization of a double filler instead of a single filler was also reported, combining not only the properties of different materials but also exploiting a synergic effect between them. For example, dual-filler HPW-g-C_3_N_4_ showed promising results; in this case, g-C_3_N_4_ serves both to stabilize HPW and to improve proton conductivity. Among the different composite membranes, the incorporation of CQD/g-C_3_N_4_ in the SPEEK membrane resulted in the highest enhancement in proton conductivity and MPD [136].

Notwithstanding these advantages, the low interaction between the polymer membrane and g-C_3_N_4_/h-BN creates agglomeration and hinders the addition of large amounts of nitrides to the PEM membranes. Moreover, the blockage of the ionic channel due to agglomeration limits the hopping of water molecules and gives rise to low proton conductivity. As reported by Huang et al. [126] and Galhot et al. [129], the addition of 0.5 wt% h-BN increases the proton conductivity of the polymer membrane, but for a h-BN content of >0.5 wt%, a decrease in proton conductivity was observed due to H-BN agglomeration in the polymer membrane. h-BN and the acidic sulfonic acid group create acid–base ion pairs, forming a continuous proton transport channel. Moreover, alkaline h-BN can promote sulfonate dissociation to expose additional proton acceptor sites. Moreover, if h-BN has proton conduction ability, its contribution is negligible compared with that of the polymer; thus, the use of excessive h-BN will only increase proton transport resistance and, thus, reduce proton conduction. In the coating method, the adhesion between nanomaterials and substrates is not sufficient, so more studies have to address the design of the thin layer and better adhesion between the two. In the case of the solution-casting method, to limit particle agglomeration, research efforts have focused on the particle distribution in the polymer solution, casting temperature and time, and the humidity and temperature of drying.

## Figures and Tables

**Table 1:** Advantages and disadvantages of the use of pristine, doped, and composite g-C3N4 and h-BN in low-temperature fuel cells.

	Cell Component	Compound	Advantages	Disadvantages
g-C_3_N_4_	Catalyst support	Pristine g-C_3_N_4_	High catalytic activity of supported catalysts, high stability,	Poor conductivity
		g-C_3_N_4_–CB and g-C_3_N_4_–rGO composites	High catalytic activity of supported catalysts, high conductivity	Moderate stability
	ORR catalyst	OMCN and 1D g-C_3_N_4_	Good ORR activity in alkaline media, high stability, high methanol tolerance	Poor ORR activity in acid media, moderate conductivity, 2e^−^ transfer
		g-C_3_N_4_–carbon composites	Good ORR activity in alkaline and acid media, High conductivity, 4e^−^ transfer	Moderate stability
		O-, S-, and P-doped g-C_3_N_4_ and g-C_3_N_4_–carbon composites	High ORR activity, close to Pt, high conductivity, 4e^−^ transfer	Moderate stability of carbon composites
		Cu-, Fe-, and Co-doped g-C_3_N_4_ and g-C_3_N_4_–carbon composites	High ORR activity, higher than Pt, high stability, 4e^−^ transfer, high methanol tolerance	Moderate stability of carbon composites
	Composite membrane	SPEEK, SPAES, and QPAEK/ mesoporous g-C_3_N_4_, F-, O-, P-, and H-doped g-C_3_N_4_, and sulfonic acid functionalized g-C_3_N_4_Double filler GO, CeO_2_, TiO_2_, Gd2Zr2O7, and HPW/g-C_3_N_4_	High proton conductivity, high stability, high fuel cell performance, used with PEM and AEM	
h-BN	Catalyst support	p-h-BN and h-BNNSs	Good activity of supported catalysts, co-catalytic activity, high durability	
	ORR catalyst	Ni(111)- and Cu(111)-supported h-BN and defective h-BNNS	High ORR activity, close to Pt, high stability	Poor conductivity
		TM-doped h-BN and h-BNNs	High ORR activity, close to Pt., 4e^−^ transfer, high stability	Only theoretical calculations
		Carbon- and graphene-doped h-BN	High ORR activity, close to Pt, 4e^−^ transfer, high methanol tolerance	
		Tri-functional h-BN/Cu/G	High ORR activity, close to Pt, 4e^−^ transfer	
	Composite membrane	Nafion, SPEEK-, SPES-, and PBI-functionalized h-BN and h-BNNSs	High proton conductivity, high stability, high fuel cell performance	Only used with PEM

## Data Availability

No new data were created or analyzed in this study.

## Abbreviations

Accelerated durability test: ADT; alkaline fuel cell: AFC; anion exchange membrane: AEM; bipolar plate: BP; boron nitride microfiber: BNMF; boron nitride nanofiber: BNNF; carbon black: CB; carbon nanofiber: CNF; carbon nanosheet: CNS; carbon nitride nanoflakelet: CNNF; catalyst layer: CL; cyclic voltammetry: CV: direct ethanol fuel cell: DEFC; degree of sulfonation: DS; direct formic acid fuel cell: DFAFC; direct methanol fuel cell: DMFC; electrochemically active surface area: ECSA; formic acid oxidation reaction: FAOR; gas diffusion layer: GDL; graphene: G; graphene nanoplatelet: GNP; graphene oxide: GO; graphene quantum dot: GQD; graphitic carbon nitride: g-C_3_N_4_; hexagonal boron nitride: h-BN; hexagonal boron nitride nanosheet: h-BNNS; hollow mesoporous g-C_3_N_4_ nanosphere/3D graphene composite:HMCN-3G; phosphotungstic acid: HPW; mass activity: MA; maximum power density: MPD; membrane electrode assembly: MEA; mesoporous carbon nitride: MCN; methanol oxidation reaction: MOR; multimodal porous carbon: MPC; multiwalled carbon nanotube: MWCNT; Nafion functionalized boron nitride: NBN; open circuit voltage: OCV; ordered mesoporous carbon: OMC; oxygen reduction reaction: ORR; perfluorosulfonic acid: PFSA; polyaniline: PANI; polybenzimidazole: PBI; polydopamine: PDA; proton exchange membrane: proton exchange membrane fuel cell: PEMFC; PEM; quaternary poly(arylene ether ketone): QPAEK; reduced graphene oxide: relative humidity: RH; rGO; specific activity: SA; strong metal-support interaction: SMSI; sulfonated poly (arylene ether sulfone): SPAES; sulfonated poly(ether ketone ether): SPEEK; sulfonated poly(ether sulfone): SPES; transition metal: TM.
